# Evolutionary analysis of a complete chicken genome

**DOI:** 10.1073/pnas.2216641120

**Published:** 2023-02-13

**Authors:** Zhen Huang, Zaoxu Xu, Hao Bai, Yongji Huang, Na Kang, Xiaoting Ding, Jing Liu, Haoran Luo, Chentao Yang, Wanjun Chen, Qixin Guo, Lingzhan Xue, Xueping Zhang, Li Xu, Meiling Chen, Honggao Fu, Youling Chen, Zhicao Yue, Tatsuo Fukagawa, Shanlin Liu, Guobin Chang, Luohao Xu

**Affiliations:** ^a^Integrative Science Center of Germplasm Creation in Western China (CHONGQING) Science City, Key Laboratory of Freshwater Fish Reproduction and Development (Ministry of Education), Key Laboratory of Aquatic Science of Chongqing, School of Life Sciences, Southwest University, Chongqing 400715, China; ^b^Fujian Key Laboratory of Developmental and Neural Biology, College of Life Sciences, Fujian Normal University, Fuzhou 350117, China; ^c^Fujian-Macao Science and Technology Cooperation Base of Traditional Chinese Medicine-Oriented Chronic Disease Prevention and Treatment, Innovation and Transformation Center, Fujian University of Traditional Chinese Medicine, Fuzhou 350108, China; ^d^Gansu Key Laboratory of Protection and Utilization for Biological Resources and Ecological Restoration, College of Life Sciences and Technology, Longdong University, Qingyang, Gansu Province 745000, China; ^e^Joint International Research Laboratory of Agriculture and Agri-Product Safety, the Ministry of Education of China, Yangzhou University, Yangzhou 225009, China; ^f^Key Laboratory of Animal Genetics and Breeding and Molecular Design of Jiangsu Province, College of Animal Science and Technology, Yangzhou University, Yangzhou 225009, China; ^g^Institute of Oceanography, Minjiang University, Fuzhou 350108, China; ^h^Department of Neuroscience and Developmental Biology, University of Vienna, Vienna 1090, Austria; ^i^Key Laboratory of Ministry of Education for Coast and Wetland Ecosystems, College of the Environment and Ecology, Xiamen University, Xiamen 361102, China; ^j^BGI-Shenzhen, Shenzhen 518083, China; ^k^Aquaculture and Genetic breeding laboratory, Freshwater Fisheries Research Institute of Fujian, Fuzhou 350002, China; ^l^Department of Cell Biology and Medical Genetics, International Cancer Center, and Guangdong Key Laboratory for Genome Stability and Disease Prevention, Shenzhen University School of Medicine, Guangdong, 518054, China; ^m^Graduate School of Frontier Biosciences, Osaka University, Suita, Osaka 565-0871, Japan; ^n^Department of Entomology, China Agricultural University, Beijing 100193, China

**Keywords:** chicken genome, dot chromosome, centromere, chromosome evolution

## Abstract

Microchromosomes are prevalent in nonmammalian vertebrates [P. D. Waters *et al.*, *Proc. Natl. Acad. Sci. U.S.A.* **118** (2021)], but a few of them are missing in bird genome assemblies. Here, we present a new chicken reference genome containing all autosomes, a Z and a W chromosome, with all gaps closed except for the W. We identified ten small microchromosomes (termed dot chromosomes) with distinct sequence and epigenetic features, among which six were newly assembled. Those dot chromosomes exhibit extremely high GC content and a high level of DNA methylation and are enriched for housekeeping genes. The pericentromeric heterochromatin of dot chromosomes is disproportionately large and continues to expand with the proliferation of satellite DNA and testis-expressed genes. Our analyses revealed that the 41-bp CNM repeat frequently forms higher-order repeats (HORs) at the centromeres of acrocentric chromosomes. The centromere core regions where the kinetochore attaches often encompass telomeric sequence (TTAGGG)n, and in a one of the dot chromosomes, the centromere core recruits an endogenous retrovirus (ERV). We further demonstrate that the W chromosome shares some common features with dot chromosomes, having large arrays of hypermethylated tandem repeats. Finally, using the complete chicken chromosome models, we reconstructed a fine picture of chordate karyotype evolution, revealing frequent chromosomal fusions before and after vertebrate whole-genome duplications. Our sequence and epigenetic characterization of chicken chromosomes shed insights into the understanding of vertebrate genome evolution and chromosome biology.

## Significance

Chicken is one of the most important vertebrate model organisms, yet its genome is far from complete. This study generated the complete sequence of a chicken genome, uncovering six chromosome models absent in previous genome assemblies. Ten small microchromosomes evolved distinct genomic and epigenetic features, unlike any other vertebrate chromosomes but remain stable and conserved in birds. Most chicken centromeres were found to contain higher-order repeats (HORs), resembling the centromeric organization in primates. The complete chicken chromosome models are useful to reconstruct the karyotype of the vertebrate ancestor. We reveal the evolutionary trajectory of chromosome changes from ancestral chordate to early vertebrate and Amniota through frequent fusion events before and after whole-genome duplications.

Chicken is one of the most important vertebrate model organisms, and its genome is widely used for study in vertebrate evolution and avian biology (1–3). The first draft chicken genome was sequenced from an inbred line UCD001 in 2004 with shotgun reads, combined with reads from bacterial artificial chromosomes (BACs) and fosmids (4) and had been subsequently improved (5). The current reference genome (GRCg6a) was created by Genome Reference Consortium (GRC) using the same DNA source but employing PacBio single-molecular long reads (6). The Vertebrate Genome Project (VGP) has also released a genome assembly (bGalGal1.mat.broiler.GRCg7b) from a female chicken using a trio-binning approach (7). Various maps have been used to improve chromosomal anchoring, including a consensus linkage map, East Lansing map, and radiation hybrid map (4). However, although karyotype analyses revealed that the chicken genome contains 2n = 78, thus far only 32 autosomes plus two sex chromosomes (Z and W) were assembled, indicating six chromosomes missing.

A typical avian genome contains 10 pairs of macrochromosomes and ~30 pairs of microchromosomes that are evolutionarily stable during avian diversification (8, 9) and is thought to resemble ancestral vertebrate karyotype (10–12). It has been recently established that Amniota microchromosomes are stable (13) and have a chordate origin (14), likely formed due to asymmetric sequence losses following vertebrate whole-genome duplications (WGD) (15, 16). The six missing chromosomes in chicken genomes are all microchromosomes, expected to be gene-dense like other microchromosomes (17). A recent chicken pan-genome construction with PacBio reads has identified more than 1,000 novel genes that are enriched on microchromosomes (18). Creating a new reference chicken genome with all chromosome models assembled will reveal a complete gene repertoire and a full picture of vertebrate karyotype evolution.

The use of Nanopore ultralong reads and PacBio HiFi reads permits telomere-to-telomere genome assembly (19, 20). The HiFi reads can resolve complex regions (21) while the ultralong reads can assist to resolve tandem duplications (22). The combination of those two sequencing technologies has successfully completed the human genome (23), which provides a promising strategy to produce finished genomes for other species (24). Here, we present a complete chicken genome with all chromosomes assembled and all gaps closed except for the W chromosome.

## Results

### Toward the Complete Sequence of a Chicken Genome.

**Fig. 1.**
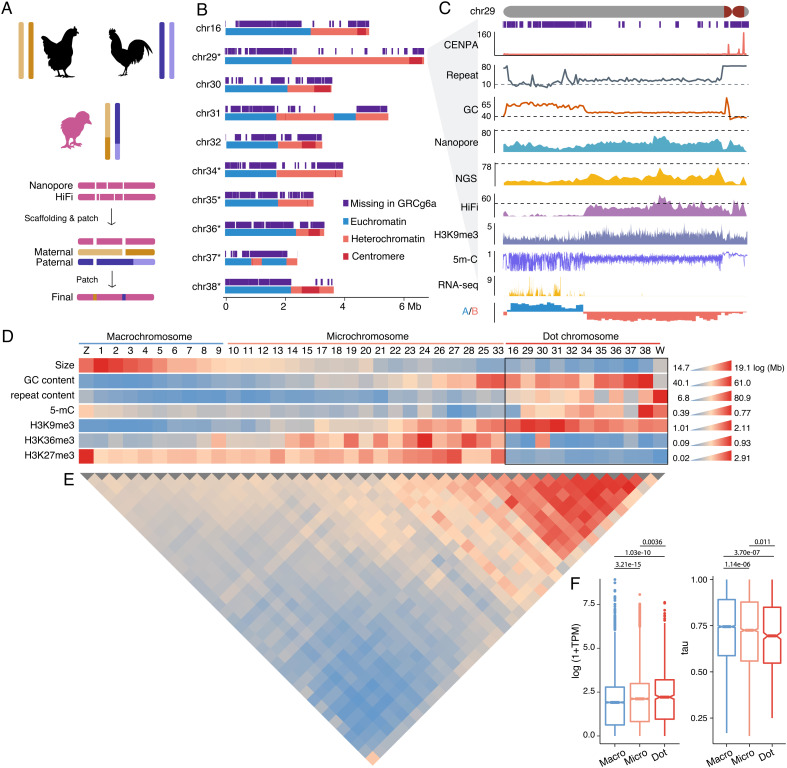
A complete chicken genome with 10 dot chromosomes. (*A*) A trio-based genome assembly pipeline. Rounded rectangles represent contigs. Paternal and maternal contigs were used to fill gaps in the primary contigs. (*B*) The dot chromosomes are in general composed of a euchromatic part and a heterochromatic part. The asterisks denote newly assembled chromosome models. (*C*) A zoom-in view for chr29, showing CENP-A and H3K9me3 binding, coverage of Nanopore ultralong, HiFi, NGS (BGISEQ-500, dashed lines indicate genomic average), gene expression (RNA-seq read counts in 1 kb windows), 5-mC levels, and A/B compartments. (*D*) The heatmap shows the chromosomal sizes (log-transformed), GC content, repeat content, chromosome-wide 5-mC levels, and ChIP/input ratios for H3K9me3, H3K36me3, and H3K27me3. (*E*) Interchromosomal interaction frequency measured using Hi-C data. (*F*) Dot chromosomes have a lower Tau value, i.e., lower level of tissue specificity but a higher expression level. *P* values were calculated using the Wilcoxon signed-rank tests.

We employed a trio-sequencing approach (25) to assemble the diploid genome of a female chick (Huxu breed, Fig. 1*A*). We generated ~80× ultralong Nanopore and ~52× HiFi reads and both produced highly continuous genome assemblies (*SI Appendix*, Tables S1 and S2). We used HiFi-based contigs to replace sequences and fill gaps in the Nanopore-based backbone assembly (Fig. 1*A*). The integration of the two datasets resulted in contigs already at chromosome-scale, with only 26 gaps remaining. Those gaps are mostly embedded within long arrays of satellite DNA or simple repeats.

We further partitioned ultralong and HiFi reads derived from parental and maternal haploids and produced two haploid assemblies using the same approach described above (Fig. 1*A*). The paternal and maternal contigs are expected to resolve complex regions that often lead to gaps in haploid (primary) genome assembly. Indeed, the remaining gaps, except for those on the W chromosome, are all resolved by one of the haploid assemblies or both (Fig. 1*A*). For instance, a ~3 Mb subtelomeric sequence at the 5′ end of chromosome 1 (chr1) was assembled into four contigs in the primary assembly but a single contig in both paternal and maternal haploid assembly (*SI Appendix*, Fig. S1).

**Fig. 2.**
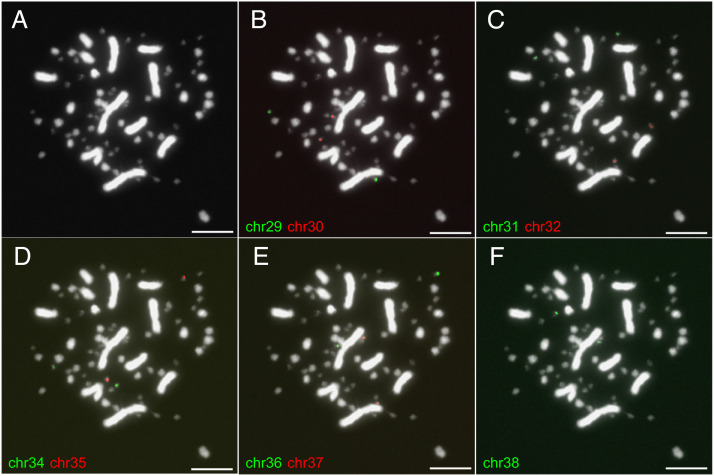
FISH verification for dot chromosomes. (*A*) The karyotype of a chicken cell. (*B*–*F*) The probes of nine dot chromosomes bind to different chromosomes in the same cell. The probes are composed of unique oligonucleotides (Dataset S2) and chromosome-specific amplicons (Fig. 5*F*). Chromosome 16 has been verified by previous studies (27). The scale for the white bar: 10 μm.

The final assembly (GGswu1) contains 38 autosomes, a Z and a W chromosome (*SI Appendix*, Fig. S2), consistent with the known female karyotype (Fig. 2*A*) (26). Compared with the reference genome GRCg6a, the total length increases by 50.4 Mb to 1.1 Gb, mainly attributed to the addition of satellite DNA or segments with GC content larger than 55% (GC plateaus, *SI Appendix*, Table S3). Satellite DNA occupied 5.0% of the new assembly compared to 3.0% in GRCg6a. Importantly, our new assembly contains six chromosomal models (chr29, chr34 to 38) that are absent in GRCg6a. The homologous sequences of newly assembled chromosomes are scattered in the unanchored scaffolds or incorrectly anchored to chr31 and chr33 in GRCg6a (*SI Appendix*, Fig. S3). The GRCg7b assembly has proposed seven new chromosome models homologous to the six new ones in GGswu1, but their total size is only 13.4 Mb compared with 40.4 Mb in GGswu1 (*SI Appendix*, Table S4). We mapped more than 1,000 BAC clones against GGswu1 and found that 99.9% of the BAC sequences were aligned, and the one-to-one alignments have an average identity of 99.2% (*SI Appendix*, Table S5).

Approximately 44.6% of heterochromatic parts of the newly uncovered chromosomes are absent in GRCg6a, but unexpectedly 77.6% of the euchromatic parts carrying coding genes were absent (Fig. 1*B* and *SI Appendix*, Fig. S4 and Table S6). This is likely because the euchromatin has a much higher GC content than heterochromatin (58.7% vs. 51.4%), despite a lower repeat content (29.5% vs. 61.9%, *SI Appendix*, Fig. S5). The extremely high GC content makes the euchromatin inaccessible for HiFi or next-generation sequencing (NGS) but fortunately less so for Nanopore sequencing (Fig. 1*C* and *SI Appendix*, Fig. S4).

### The Chicken Genome Contains 10 Dot Chromosomes.

The six newly assembled chromosomes all have a small size (on average 3.9 Mb), much smaller than other microchromosomes (~10.2 Mb), but exhibit a much higher GC content and repeat content, a higher level of chromosome-wide DNA methylation (5 mC), and H3K9me3 histone modifications but depletion of H3K36me3 and H3K27me3 (Fig. 1*D* and *SI Appendix*, Table S7). Four previously partially assembled chromosomes (chr16, 30 to 32) share similar features (Fig. 1*D* and *SI Appendix*, Table S7). Collectively, we termed those 10 chromosomes (chr16, 29, 30 to 32, 34 to 38) as dot chromosomes based on their morphology and heterochromatic nature. This term does not necessarily imply common features with the *Drosophila* dot chromosome (28). Other authors have used the term “D group” to refer to those dot-like chromosomes (26). The average size of the dot chromosome is 4.0 Mb, in line with the estimated size by a pulse electrophoresis method for the smallest chicken microchromosomes (3.4 to 4.8 Mb) (29). The ten dot chromosomes collectively account for only 3.7% of the chicken genome. We synthesized unique oligonucleotides across the euchromatin and amplified chromosome-specific duplicated genes (see the section ‘*Testis-Expressed Amplicons in pericentromeric heterochromatin (PCH)*’) as probes to verify the dot chromosomes. As a result, each chromosome-specific probe binds to one pair of chromosomes (Fig. 2).

The dot chromosomes typically have a highly compartmentalized chromatin organization, with the compact euchromatin occupying a part of the long-arm, and centromeric and PCH on the other part of the chromosomes (Fig. 1 *B* and *C* and *SI Appendix*, Fig. S4). In line with the abovementioned disparity in GC and repeat content, the heterochromatin and euchromatin parts of the dot chromosomes display distinct epigenetic features, including an extremely high methylation level for the heterochromatin (Fig. 1*C* and *SI Appendix*, Fig. S4). Similar to microchromosomes (30, 31), the dot chromosomes show intensive interchromosomal interactions between themselves (Fig. 1*E*), consistent with their physical clustering in the nuclear center (32) and are mainly driven by interactions between euchromatin (*SI Appendix*, Fig. S6). Despite intensive interchromosomal interactions, the dot chromosomes clearly show their own chromosomal territories (*SI Appendix*, Fig. S2*B*), supporting isolated assembly of chromosome models.

There is no sharp borderline between macrochromosomes and microchromosomes or between microchromosomes and dot chromosomes (Fig. 1*D* and *SI Appendix*, Fig. S7). For instance, chr25 and chr33 share some features with dot chromosomes, including small sizes (~4.1 Mb) and high GC contents (Fig. 1*D*). Their repeat content, epigenetic features (5-mC, H3K36me3 and H3K27me3), and the intensity of interchromosomal interactions, on the other hand, make them distanced from dot chromosomes, though the H3K9me3 landscape appears to be similar (Fig. 1*D* and *SI Appendix*, Table S7).

The gene density of dot chromosomes is much higher than that of macrochromosomes, but similar to microchromosomes, with an average gene number of 89. The newly assembled or completed dot chromosomes supplied additional 307 genes previously missed. Collectively, dot-chromosome genes have higher expression than microchromosomes or macrochromosomes and are more widely expressed (Fig. 1*F* and *SI Appendix*, Fig. S8), thus more likely housekeeping genes.

### Reconstruction of Vertebrate Ancestral Karyotype.

**Fig. 3.**
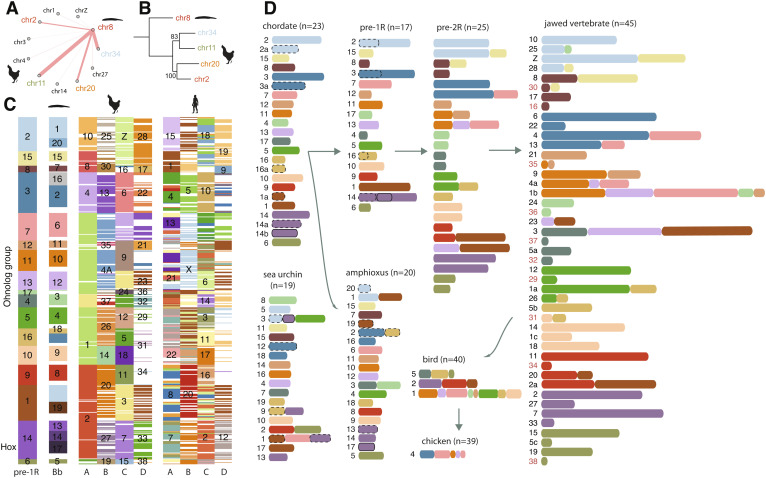
Chordate karyotype evolution. (*A*) Genes from amphioxus chromosome 8 are primarily homologous with those from four chicken chromosomes. The thickness of connecting lines indicates the relative abundance of homologous genes for chicken chromosomes. (*B*) Maximum-likelihood phylogeny of concatenated homologous genes from the homologous chromosomes in (*A*). Bootstrapping values are shown at the nodes. (*C*) 478 ohnolog groups that have at least three ohnologs in chicken. Each row represents one ohnolog group. The numbers indicate the chromosomes or ancestral linkage groups. White colors imply the absence of ohnologs. Chicken and human have up to four homologous chromosomes (*A*–*D*) for each amphioxus (Bb) chromosome. (*D*) The reconstructed evolutionary history of vertebrate chromosomes. The IDs of dot chromosomes are colored in red in the jawed vertebrate panel.

We used the complete chromosomal assembly of the chicken genome to reconstruct the full picture of vertebrate karyotype evolution. Our recent efforts have demonstrated that most amphioxus chromosomes are homologous with four different chicken chromosomes (1:4 relationships) due to two-round (2R) vertebrate WGD, except for six chromosomes with 1:3 relationships (15). Here, we rediscovered the 1:4 relationships for those six 1:3 cases by finishing the six new dot-chromosomes (Fig. 1*A* and *SI Appendix*, Fig. S9). For example, genes on amphioxus chr8 are homologous with those on chicken chr2, chr11, chr20, and chr34 (Fig. 3 *A* and *B*), among which chr34 is a newly uncovered dot-chromosome. Assigning homologous chicken chromosomes in turn led to the identification of 478 homologous gene (ohnolog) groups that have at least three ohnolog members in chicken (Fig. 3*C* and Dataset S1). Those ohnolog groups then led to the identification of 197 new ohnologs in the human genome based on chicken-human orthologous relationships (Fig. 3*C* and Dataset S1) and are helpful to corroborate the origin of microchromosomes and dot-chromosomes due to asymmetric sequence losses on one of the duplicated chromosomes following WGDs (15, 16).

While we reconstructed 17 ancestral chromosomes prior to the vertebrate first WGD (1R), in agreement with other studies (33, 34), we found that the chordate ancestral karyotype likely had 23 chromosomes, and they experienced six fusions in the branch leading to proto-vertebrate and three independent fusions in the branch to Cephalochordata (Fig. 3*D*). We further revealed drastic chromosomal changes following WGD, including nine post-1R fusions and five post-2R fusions (Fig. 3*D*), largely agreeing with a recent study (16). The common ancestor of jawed vertebrates likely had 45 pairs of chromosomes which experienced only five fusion events over ~400 My of evolution leading to the bird common ancestor inferred to have 40 chromosome pairs. Those five fusions led to the formation of bird chr1, chr2, and chr5 which are all macrochromosomes.

### Origin and Diversity of Chicken Centromeres.

**Fig. 4.**
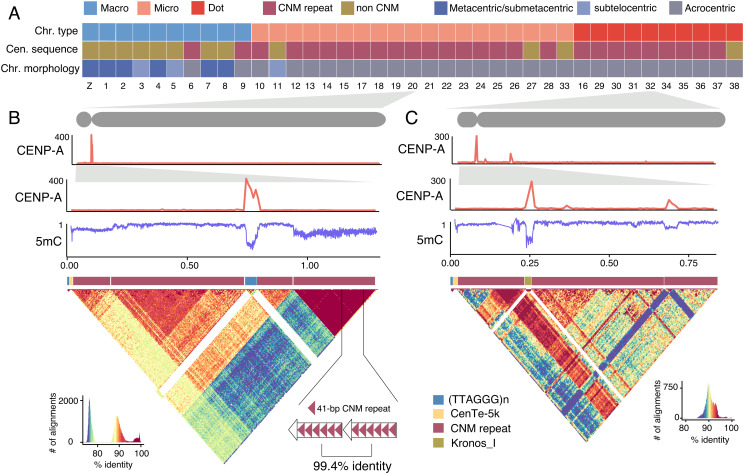
The sequences and epigenetics of chicken centromeres. (*A*) The type of centromeric repeat and the type and morphology of chromosomes. Non-CNM repeats include chromosome-specific tandem or non-tandem repeat, simple repeats, and TEs. (*B* and *C*) The ChIP/input ratio for CENP-A ChIP-seq, and 5 mC levels (0 to 1) estimated with Nanopore reads on the centromeres of chr20 and chr32. The CENP-A peaks have the lowest 5-mC levels. The heatmaps show the pair-wise sequence identity (%) between 4-kb sequences. CNM repeats are divergent from each other but often form HORs.

Chicken macrochromosomes tend to be metacentric or submetacentric (8 out of 10) whose unique centromeric repeats are largely known (35), in contrast to microchromosomes or dot-chromosomes that are mostly (28 out of 29) acrocentric (Fig. 4*A*), with their centromeric sequences remaining elusive (36). This is in part due to the difficulty in resolving the short-arm sequences of acrocentric chromosomes (23). With the new chicken assembly, we show that the acrocentric centromeres are 0.2 to 0.8 Mb long and are almost always associated with tandem arrays of a 41-bp repeat (Fig. 4*A* and *SI Appendix*, Figs. S10–S12) known as the CNM repeat (*SI Appendix*, Fig. S13) (35, 37–39). The CNM repeat-associated centromeres are also present in two unusually acrocentric macrochromosomes (chr6 and chr9, *SI Appendix*, Fig. S10) (38), suggesting that the morphology (centromere position), rather than the size of chromosomes, determines the composition of centromeric sequences.

Given that chordate ancestral chromosomes were likely all acrocentric (15), we hypothesize that CNM repeat-associated acrocentric centromeres are ancestral and stable during avian evolution. On the contrary, sub/metacentric macrochromosomes usually contain chromosome-specific tandem repeats in the centromeres (35) that are likely derived and were formed upon chromosomal fusions (40) following WGDs (Fig. 3*D*). Supporting this, the centromere of chr1 locates near a fusion point and is only 2.2 Mb away from a large interstitial telomeric repeat (*SI Appendix*, Fig. S14) (38), suggesting its likely formation following chromosomal fusions and the loss of CNM repeats. The disparity in centromere sequence composition and property between macrochromosomes and microchromosomes or dot chromosomes may play a role in driving their spatial segregation in the interphase nucleus (41).

In 19 acrocentric microchromosomes and dot chromosomes, the short arm encompasses merely a 5-kb conserved sequence (CenTe_5k) apart from the telomeric repeats (TTAGGG)n and CNM clusters (Fig. 4 *B* and *C* and *SI Appendix*, Table S8). The binding sites of CENP-A that define kinetochores and centromere cores are usually not on the body of CNM clusters, but at or close to their boundaries. In seven chromosomes, the centromere core sequences consist of (TTAGGG)n embedded within CNM tandem arrays, and in eight chromosomes, other simple repeats, such as (GCCTT)n, were recruited at centromere cores (*SI Appendix*, Figs. S11 and S12). Curiously, on the dot chromosome 32, the centromere core sequence consists of three tandemly arrayed full-length Kronos_I, an endogenous retrovirus (ERV) element (Fig. 4*C*). In spite of the differences in sequence composition or origin, the centromere cores show a deficient level of DNA methylation (Fig. 4 *B* and *C* and *SI Appendix*, Figs. S10–S12), though in some chromosomes the patterns are obscure, possibly due to centromere shifts (42).

The human centromeres are characteristic of higher-order repeats (HORs) (43) that evolved through “layered expansions” (44). Similarly, we discovered that the CNM monomer frequently forms HORs in acrocentric chromosomes in spite of their large intra- and inter-chromosomal divergence (Fig. 4*B*). The CNM HOR units contain 26 to 98 copies of the CNM repeat, and each HOR cluster contains 5 to 162 HOR units. In seven acrocentric chromosomes (chr9–10, 12–14, 17, 20), the active HORs sit at the distal end of centromeres, suggesting the expansion of HORs in the direction away from short-arm telomeres (Fig. 4*B* and *SI Appendix*, Figs. S10–S12), while in others (e.g., chr32) the active HORs flank the centromere cores (Fig. 4*C*). These two modes (outward and inward) of tandem-repeat expansion also apply to macrochromosome centromeres, with younger expansions at the centromere edge in, for example, chr1, but at the centromere centers in chr2 to 4 (*SI Appendix*, Fig. S10).

### Testis-Expressed Amplicons in PCH.

**Fig. 5.**
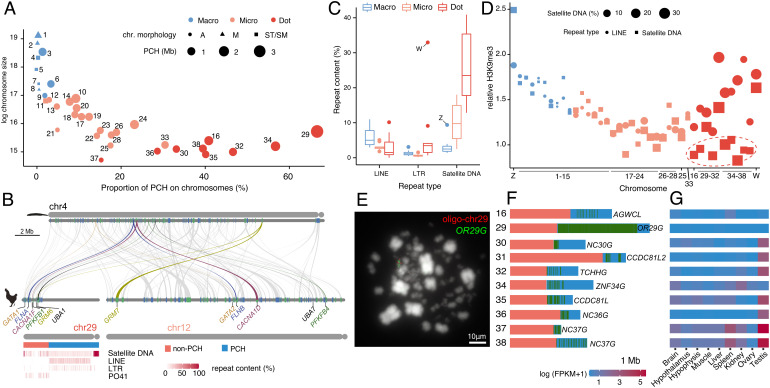
The expansion of PCH and amplicons. (*A*) The dot-chromosomes have larger PCH and the proportion of PCH is higher relative to microchromosomes and macrochromosomes. (*B*) Chicken chr12 and chr29 are homologous with amphioxus chr4. Six ohnolog pairs between chr29 and chr12 are highlighted. The PCH region of chr29 is rich in TEs while the non-PCH region is depleted of retrotransposons but is enriched for the PO41 repeats (37). (*C*) Satellite DNA is the most abundant repeat sequence on dot-chromosomes. (*D*) In macrochromosome and microchromosomes the relative H3K9me3 levels are similar for LINEs and satellite DNA while in dot-chromosomes the satellite DNA tends to have a lower relative H3K9me3 level (red dashed ellipse). (*E*) The PCH of dot chromosomes contains a large number of multicopy genes. Each green vertical bar represents one copy. (*F*) OR29G was detected in the PCH of chr29 while the synthesized oligonucleotides (oligo-chr29) were detected in the other part of chr29. (*G*) The expression of multicopy genes is testis-biased for seven out of the 10 dot chromosomes.

The morphology of chromosomes also determines the extent of PCH expansion (20). In particular, PCH is smaller in non-acrocentric chromosomes but expands to a larger size in acrocentric chromosomes (Fig. 5*A*). As a consequence, PCH occupies ~40.8% of dot chromosomes, compared to ~12.3% in microchromosomes and ~1.0% in macrochromosomes (Fig. 5*A*). In one extreme case, the proportion of PCH reaches 66.6% on chr29, leaving coding genes crowding in a small euchromatic region depleted of transposable elements (TEs) (Fig. 5*B*). Satellite DNA is the major component of dot-chromosome repeat sequences (Fig. 5*C*) and shows a lower level of H3K9me3 modifications compared to LINEs and LTRs (Fig. 5*D*). We propose that the less repressed satellite DNA is likely a major contributor to PCH expansion in dot chromosomes.

The repetitive PCH regions in dot-chromosomes, however, surprisingly contain a large number of duplicated protein-coding genes or lncRNAs (Fig. 5*F*). For instance, on chr29, an olfactory receptor gene (named *OR29G*) has 386 intact copies (Fig. 5 *E* and *F*). Interestingly, in seven out of 10 dot-chromosomes, the amplified genes are strongly testis-biased or testis-specific (Fig. 5*G*). Those testis-expressed amplicons tend to be closer to PCH boundaries (Fig. 5 *F* and *G*) and likely are still increasing their copy numbers and invading the euchromatin.

### The W Chromosome as a Putative Dot Chromosome.

The Z and W chromosomes of birds evolved from an ordinary autosomal pair more than 100 Mya (45); while the Z chromosome remains the fifth largest chromosome, the W chromosome has experienced massive sequence loss, repeat accumulation, and extensive heterochromatinization (46). The length of the new W chromosome assembly reaches 14.2 Mb (*SI Appendix*, Fig. S15), double the size of the one of GRCg6a, and 55% larger than that of GRCg7b. The centromere has also been assembled with a strong CENP-A signal at ~7 Mb, closely linked to CNM and (TTAGGG)n repeats (*SI Appendix*, Fig. S16). Repetitive sequences occupy 87% of the W chromosome, including 4.9 Mb satellite DNA which is the most abundant repeat class. Interestingly, all sequence gaps on the W chromosomes are flanked by large arrays of satellite DNA (*SI Appendix*, Fig. S16), suggesting that some satellite DNA is still missing in the assembly. The most abundant satellite DNA is a 21-bp element (TTTTCnnnnnGAAAAnnnnnn) named sate-21, accounting for 19.7% of the W chromosome sequence. The sate-21 sequences contain a conserved dyad symmetry, similar to that in CNM (39), and they form divergent HORs (*SI Appendix*, Fig. S16).

Similar to some dot-chromosome amplicons, *HINT1W* thought to be the only multicopy gene on the chicken W chromosome was amplified through the expansion of satellite DNA. We identified a 5,648-bp sequence containing the complete sequence of *HINT1W* that has been tandemly duplicated 52 times (*SI Appendix*, Fig. S16). This satellite DNA forms two distinct clusters, suggesting two waves of *HINT1W* amplification (*SI Appendix*, Fig. S16). This region constantly shows a high GC content (53.6%), possibly driven by GC-biased gene conversion between *HINT1W* copies. In addition to *HINT1W*, we discovered another two multicopy genes: two copies of *RPL17* and three copies of *TXNL1* (*SI Appendix*, Fig. S16).

Apart from differences in gene and sequence composition between the Z and W chromosomes, the two also displayed apparent epigenetic disparity. For instance, H3K27me3 modifications are sparse on the W chromosome, but are significantly enriched on the Z chromosome (Fig. 1*D*). On the contrary, the W chromosome has the highest level of DNA methylation; the Z chromosome has a relatively lower methylation level though higher than other macrochromosomes and contains a ~9-Mb hypermethylated region (*SI Appendix*, Fig. S17) encompassing testis-expressed amplicons (47).

## Discussion

A complete chicken genome (except for the W) is achieved thanks to the Nanopore ultralong and PacBio HiFi sequencing technology and a trio-binning strategy (48). The capacity of Nanopore sequencers in sequencing through high GC content regions (49, 50) allows the assembly of six dot chromosomes missing in older versions of chicken genomes. The dot chromosomes are typically acrocentric, largely compartmentalized into two parts: a compact and gene-rich euchromatin region and a hypermethylated PCH region. This compartmentalized chromatin organization, in particular a large portion of PCH, together with extremely small size (~4 Mb), makes dot chromosomes distinct from microchromosomes. Like microchromosomes, dot chromosomes evolved from ancestral vertebrate chromosomes, only losing much more genes and sequences. It has been inferred that saurischian dinosaur already had a small genome (51) and large chromosome number (52), therefore likely possessing microchromosomes, but it is unclear whether dot chromosomes had evolved. It is possible that dot chromosomes evolved as early as in Archelosauria but were fused with macrochromosomes or microchromosomes in turtles and crocodiles (12) while persisted in modern birds and possibly dinosaurs. Studying the evolutionary history of homology of bird dot chromosomes in turtles and crocodiles can potentially reveal the origin of and evolutionary fate of dot chromosomes.

## Materials and Methods

### Genome Sequencing.

We collected a chicken (Huxu breed) trio from a local breeder in Huizhou, Guangdong. The F1 female chick was killed for DNA extraction and long-read sequencing. For each ultralong Nanopore library, approximately 8 to 10 μg gDNA was size-selected (>50 kb) with SageHLS HMW library system (Sage Science) and processed using the Ligation sequencing 1D kit (SQK-LSK109, Oxford Nanopore Technologies). About 800 ng DNA libraries were constructed and sequenced on the Promethion platform at Grandomics Biosciences (Wuhan, China). For HiFi (CCS) sequencing, SMRTbell target size libraries were constructed according to PacBio’s standard protocol (Pacific Biosciences) using the 15-kb preparation solutions. A total amount of 15 μg DNA per muscle sample was used for the DNA library preparations. Sequencing was performed on a PacBio Sequel II instrument with Sequencing Primer V2 and Sequel II Binding Kit 2.0 at Grandomics. To prepare the Hi-C library, fixed muscle tissue was frozen in liquid nitrogen and ground to powder before resuspending in nuclei isolation buffer to obtain a suspension of nuclei. The purified nuclei were digested with 100 units of HindIII and marked by incubating with biotin-14-dCTP. The ligated DNA was sheared into 300 to 600-bp fragments and then was blunt-end repaired and A-tailed, followed by purification through biotin–streptavidin-mediated pull-down. The Hi-C libraries were then quantified and sequenced using the Illumina Hiseq platform (Illumina, San Diego).

### Nanopore Direct RNA Sequencing.

Total RNA from brain and spleen tissues was extracted by grinding tissue in TRIzol reagent (TIANGEN) on dry ice and processed following the protocol provided by the manufacturer. The direct RNA libraries were prepared using the Direct RNA Sequencing Kit SQK-RNA002 (Oxford Nanopore Technologies) according to the manufacturer’s protocol. Briefly, the poly-A RNAs were enriched using the NEBNext® Poly(A) mRNA Magnetic Isolation Module (CAT#E7490, NEB) with NEBNext Magnetic Oligo (dT)_25_ Beads. Next, 100 to 500 ng of the poly-A enriched RNAs were ligated to the reverse transcriptase adaptor using T4 DNA ligase, followed by reverse transcription. The reverse-transcribed RNAs were ligated to the sequencing adaptor and were purified using Agencourt RNAClean XP beads (Beckman Coulter). Finally, two libraries (for brain and spleen) were constructed and sequenced on two different R9.4.1 FlowCells using the PromethION sequencer (ONT, UK) at Grandomics Biosciences (Wuhan, China).

### Genome Assembly.

We used Nextdenovo (v2.4.0) to assemble the Nanopore reads into primary contigs (ont_pri) with default parameters. The contigs were then polished by the reads with Nextpolish (v1.3.1) (53). To assemble the diploid contigs, Nanopore reads were partitioned into parental and maternal using the trio mode in canu (v2.1) (25). The Nextdenovo–Nextpolish pipeline was then applied to the maternal and paternal reads, respectively, to assemble the maternal (ont_mat) and paternal (ont_pat) contigs. For PacBio HiFi reads, hifiasm (0.16.0-r369) (54) with default parameter was used to assemble the primary contigs (hifi_pri). When the lists of paternal and maternal short-reads were supplied, hifiasm was used to produce paternal (hifi_pat) and maternal (hifi_mat) contigs. We aligned hifi_pri against ont_pri and replaced the homologous sequences in ont_pri with hifi_pri sequences; when a hifi_pri contig spans the ends of two ont_pri contigs, we manually joined the ont_pri contigs, producing the first version of primary contigs (pri.v1). Following this pipeline, we produced the replaced and gap-filled contigs for paternal (pat.final) and maternal (mat.final) genomes. Finally, we used pat.final and mat.final to fill gaps in the pri.v1 assembly, producing pri.v2 (GGswu1).

### Hi-C Data Analysis.

To calculate interchromosomal interactions, Hi-C read pairs were mapped to the pri.v2 assembly using Bowtie2 (2.4.4) (55), with reads uniquely mapped and having mapping quality larger than 30 kept. We then binned and normalized the alignments using HiC-Pro (v2.10.0) (56) with default parameters at 10 Kb, 40 Kb, 100 Kb, 500 Kb, and 1 Mb resolutions under ICE-normalization (57). The interchromosomal contact frequency between each chromosome pair was determined by comparing the observed Hi-C contacts between two chromosomes to the expected contacts between them following (58), using a bin size of 40 Kb. The interactions between euchromatin were estimated after excluding all pericentromeric bins. The ratios of observed vs. expected interchromosomal contacts were log2 transformed.

To visualize the Hi-C heatmap and to do scaffolding, we used a juicer (1.7.6) (59) to process Hi-C read alignments. We then visualize the Hi-C heatmap with Juicebox (1.11.08) (60) where the order and orientation of contigs can be adjusted.

### Methylation.

Nanopore reads were aligned against the reference by minimap2 (v2.24) using the “map-ont” setting. We used nanopolish (v0.13.2) (61) for detecting 5-methylcytosine bases in a CpG context. We used the script calculate_methylation_frequency from the nanopolish package to calculate methylation frequency at every called site and further summarize the methylation frequency at 500-bp windows.

### ChIP-seq.

ChIP assays were performed by Shanghai Jiayin Biotechnology Co., Ltd, according to the standard cross-linking ChIP protocol with modifications. Briefly, cells were harvested and cross-linker with 1% formaldehyde for 10 min at room temperature. After sonication, immunoprecipitation was performed with anti-histone H3K9me3 (Abcam, ab8898, 5 μg). The immunoprecipitated complex was washed, and DNA was extracted and purified by Universal DNA Purification Kit (#DP214). The ChIP-Seq library was prepared using a ChIP-seq DNA sample preparation kit (NEBNext® UltraTMII DNA) according to the manufacturer’s instructions. Extracted DNA was ligated to specific adaptors followed by deep sequencing in the Illumina Novaseq 6000 (Annoroad Gene Technology company). ChIP-seq for CENP-A (DRR018430) (62), H3K27me3 (SRR12697592) (63), and H3K36me3 (SRR15150478) (64) data was downloaded from NCBI SRA. We aligned the ChIP-seq reads with the BWA-MEM algorithm with options “-k 50 -c 1000000”. Alignment duplications were marked with sambamba (0.6.3) (65) and were filtered with samtools (view -q 30 -F 2308). We counted the reads with BEDTools genomecov (2.29.2) (66). ChIP/input ratios were calculated in 10-kb windows. To calculate relative H3K9me3 levels for repetitive sequences, we divided the ChIp/input ratios of repeats over those of unique sequences.

### Genome Annotation.

We used Trinity (2.8.4) (67) to assemble transcripts with RNA-seq data from 10 different tissues (*SI Appendix*, Table S9). Protein sequences of chicken and humans were downloaded from the RefSeq database. We used maker (2.31.10) (68) to predict gene models with evidence from both assembled transcripts and protein homology. We further used the HISAT2 (2.1.0) (69)-StringTie (2.1.1) (70) pipeline to assemble the transcripts through a genome-guided method. We performed the Augustus (3.4.0) (71) gene model training through the BUSCO (4.0.5) (72) pipeline and predicted the gene models using the trained profile. For Nanopore full-length transcriptome data, we corrected the reads with TranscriptClean (v2.0.2) (73), mapped the clean reads with minimap2 (2.21-r1071, -x splice) (74), and predicted the gene models with StringTie. For PacBio full-length transcriptome sequencing data downloaded from SRA (75), we followed the IsoSeq3 pipeline to obtain clustered and nonredundant transcripts and their alignments against the genome. The abovementioned predicted gene model or aligned transcripts were integrated by EVM (1.1.1) (76) to predict gene models. We then used the PASApipeline (2.4.1) (77) to polish the gene models with the transcripts obtained from Trinity assembly and IsoSeq3. Repeats were masked with RepeatMasker (4.1.2) using an avian repeat library (78). To determine the repeat units of tandem repeats, we divided the repetitive sequences into 30-kb windows and used TideHunter (1.4.2) (79) to predict tandem repeats. StainedGlass (v0.1) (80) was used to visualize tandem repeats.

### Gene Expression.

The RNA-seq datasets we used for analyses are shown in *SI Appendix*, Table S9. We used HiSAT2 to map raw RNA-seq reads with the options “-k 4 --max-intronlen 40000 --min-intronlen 30”. To quantify the expression level, we counted the mapped reads using featureCounts (1.6.2) (81) with options “-M -C” and calculated the TPM (transcripts per million) values. For each gene, a mean expression level across tissues was calculated. The expression breadth was measured by calculating the tau values (82). To estimate expression levels for dot-chromosomes amplicons, we calculated the mean read counts across all copies which were then normalized by the total mRNA lengths.

### Vertebrate Karyotype Evolution.

We used OrthoFinder (2.5.2) (83) to group homologous genes from chicken, zebra finch (*Taeniopygia guttata*) (7), human, spotted gar (*Lepisosteus oculatus*) (84), white-spotted bamboo shark (*Chiloscyllium plagiosum*) (85), and amphioxus (*Branchiostoma belcheri*, Bb) (15). The Bb-chicken orthologous pairs were extracted from the pairwise relationships. For each Bb chromosome, we calculated the relative abundance of homologous genes for each chicken chromosome to determine the homologous chromosomes. In each orthologous group, we extracted genes from homologous chromosomes to identify ohnologs. We followed the analyses in Huang et al. (15) to reconstruct the evolutionary history of chordate chromosomes.

### FISH Experiments.

Chromosome-specific oligo probes were developed using a previously published pipeline (86). The single-copy oligos (45 nt or 58 nt) were screened using the Chorus2 (1.1) software (87), followed by filtering for repeat sequence by applying ChorusNGSfilter.py and ChorusNGSselect.py script (−q 0.1; -p 0.9; −d 25, 45 or 58). The oligo library was synthesized by CustomArray (Genscript, Nanjing, China). A 23 nt forward primer (T7 RNA polymerase promoter sequence) and a 20 nt reverse primer were flanked by the synthetic oligo sequences (Dataset S2). PCR amplification of the library was performed to generate double-stranded DNA templates, and then the amplified products were transcribed into RNA. Labeling of the library was performed via reverse transcription of the RNA using 5′ digoxigenin-labeled primers. Unincorporated primers and the RNA template were degraded to obtain a labeled, single-stranded oligo probe.

Chromosome suspension was prepared according to a previously described method (52). The chromosome suspension was added dropwise onto glass slides, and the slide was air-dried and then kept at −20 °C until use. Oligo probes and chromosome slides were simultaneously denatured for 1 min on 70 °C hotplates prior to hybridization in a humidified chamber at 37 °C for 48 h. Slides were washed for 3 min in 2 × SSC, 10 min in 2 × SSC, and 3 min in 1 × PBS, respectively. Hybridization signals were detected with rhodamine-conjugated anti-digoxigenin (Roche Diagnostics, Basel, Switzerland) for digoxigenin-labeled probes. Then, the slides were dried and counterstained with DAPI. An Olympus BX53 epifluorescence microscope was used to observe metaphase plates with fluorescent signals that were photographed with a cooled CCD camera and visualized using cellSens Dimension 1.9 software (Olympus Corporation).

## Supplementary Material

Appendix 01 (PDF)

Dataset S01 (XLSX)

Dataset S02 (XLSX)

## Data Availability

The assemblies and raw sequencing data are available under the NCBI accession PRJNA693184. All accessions are listed in the *SI Appendix*, Table S9. The custom codes used in the study were reposited at Github (https://github.com/lurebgi/chicken-T2T) (88). All study data are included in the article and/or *SI Appendix*.
