# Supplementary material for: Evolutionary analysis of a complete chicken genome
Source: Proc Natl Acad Sci U S A. 2023 Feb 13;120(8):e2216641120. doi: 10.1073/pnas.2216641120 (PMC9974502; doi:10.1073/pnas.2216641120)
Supplement: Supplementary file 1 — Appendix 01 (PDF) [file pnas.2216641120.sapp.pdf]

PNAS

# Supporting Information for
Evolutionary analysis of a complete chicken genome.

Zhen Huang[1,2,3,15], Zaoxu Xu[1,4,15], Hao Bai[5,6,15], Yongji Huang[7], Na Kang[1], Xiaoting Ding[1], Jing Liu[8], Haoran Luo[1,9], Chentao Yang[10], Wanjun Chen[10], Qixin Guo[5,6], Lingzhan Xue[11], Xueping Zhang[2], Li Xu[2], Meiling Chen[2], Honggao Fu[2], Youling Chen[2], Zhicao Yue[12], Tatsuo Fukagawa[13], Shanlin Liu[14], Guobin Chang[5,6,*], Luohao Xu[1,*]

1. MOE Key Laboratory of Freshwater Fish Reproduction and Development, Key Laboratory of Aquatic Science of Chongqing, School of Life Sciences, Southwest University, Chongqing 400715, China.

2. Fujian Key Laboratory of Developmental and Neural Biology, College of Life Sciences, Fujian Normal University, Fuzhou 350117, China.

3. Fujian-Macao Science and Technology Cooperation Base of Traditional Chinese Medicine-Oriented Chronic Disease Prevention and Treatment, Innovation and Transformation Center, Fujian University of Traditional Chinese Medicine, Fuzhou 350108, China.

4. Gansu Key Laboratory of Protection and Utilization for Biological Resources and Ecological Restoration, College of Life Sciences and Technology, Longdong University, Qingyang, Gansu Province, 745000, China

5. Joint International Research Laboratory of Agriculture and Agri-Product Safety, the Ministry of Education of China, Yangzhou University, Yangzhou 225009, China.

6. Key Laboratory of Animal Genetics and Breeding and Molecular Design of Jiangsu Province, College of Animal Science and Technology, Yangzhou University, Yangzhou 225009, China.

7. Institute of Oceanography, Minjiang University, Fuzhou 350108, China.

8. Department of Neuroscience and Developmental Biology, University of Vienna, Vienna 1090, Austria.

9. Key Laboratory of Ministry of Education for Coast and Wetland Ecosystems, College of the Environment and Ecology, Xiamen University, Xiamen, 361102, China.

10. BGI-Shenzhen, Shenzhen 518083, China.

11. Aquaculture and Genetic breeding laboratory, Freshwater Fisheries Research Institute of Fujian, Fuzhou 350002, China.

12. Department of Cell Biology and Medical Genetics; International Cancer Center; and Guangdong Key Laboratory for Genome Stability and Disease Prevention, Shenzhen University School of Medicine, Guangdong, China.

13. Graduate School of Frontier Biosciences, Osaka University, Suita, Osaka, 565-0871, Japan

14. Department of Entomology, China Agricultural University, Beijing 100193, China.

15. Equal contribution

*Luohao Xu, Guobin Chang.

**Email:** luohaox@gmail.com (LX), gbchang@yzu.edu.cn (GC)

**This PDF file includes:**

Figures S1 to S17
Tables S1 to S10
Legends for Datasets S1 to S2
SI References

**Other supporting materials for this manuscript include the following:**

Datasets S1 to S2

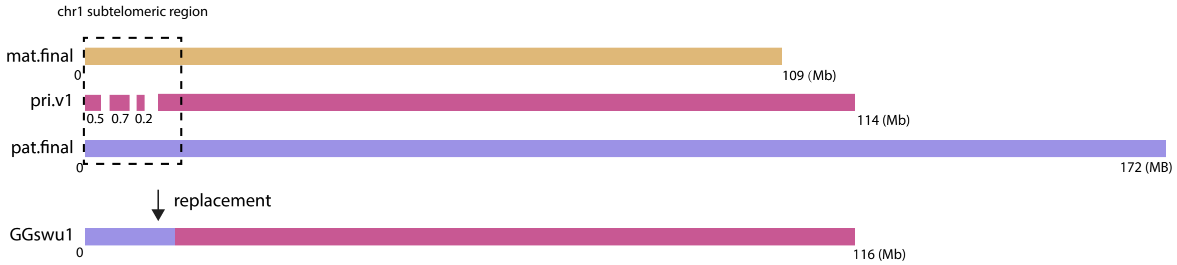


**Fig. S1. Replacement of contigs for the chr1 subtelomeric region**. Each horizontal bar represents a contig. In the assembly pri.v1 the chr1 subtelomeric region was assembled in four contigs, but the same region was assembled in a single contig in both mat.final and pat.final assembly. In GGswu1 assembly the subtolemeric region was replaced by the homologous region of pat.final.

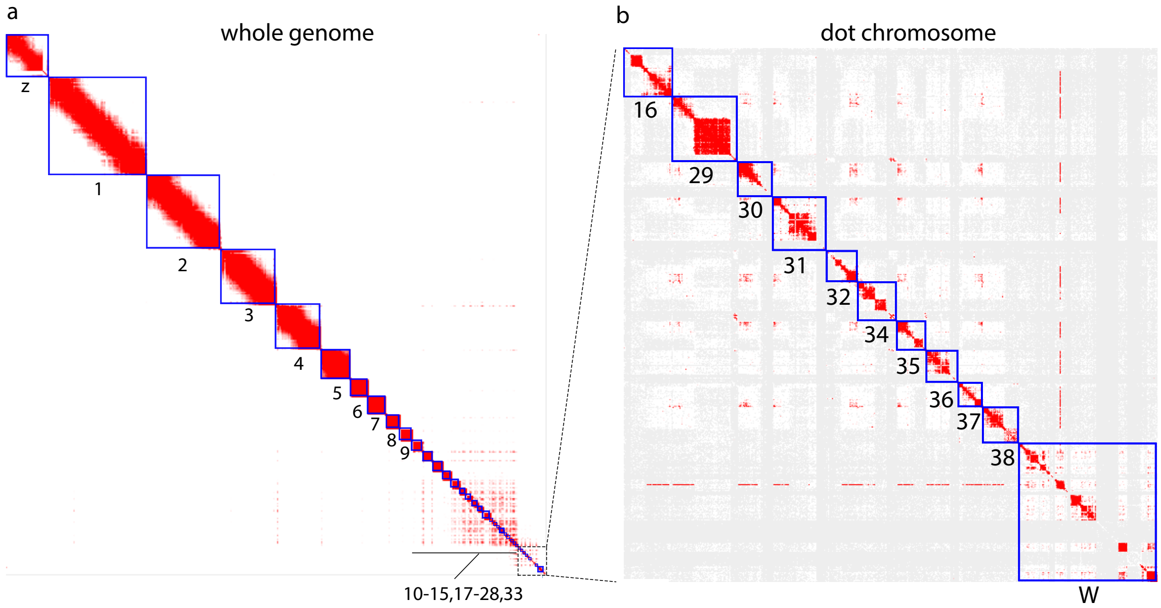


**Fig. S2. Chromosomal models in the new assembly GGswu1**. **a**) Hi-C heatmap for the whole genome visualised in Juicebox. The chromosome IDs are shown under the chromosome models. **b**) Zoom-in view for dot chromosomes. Dot-chromosomes are compartmentalized into a euchromatin and a heterochromatin region that rarely interact with each other. Large arrays of satellite DNA sequences usually are not covered by Hi-C reads.

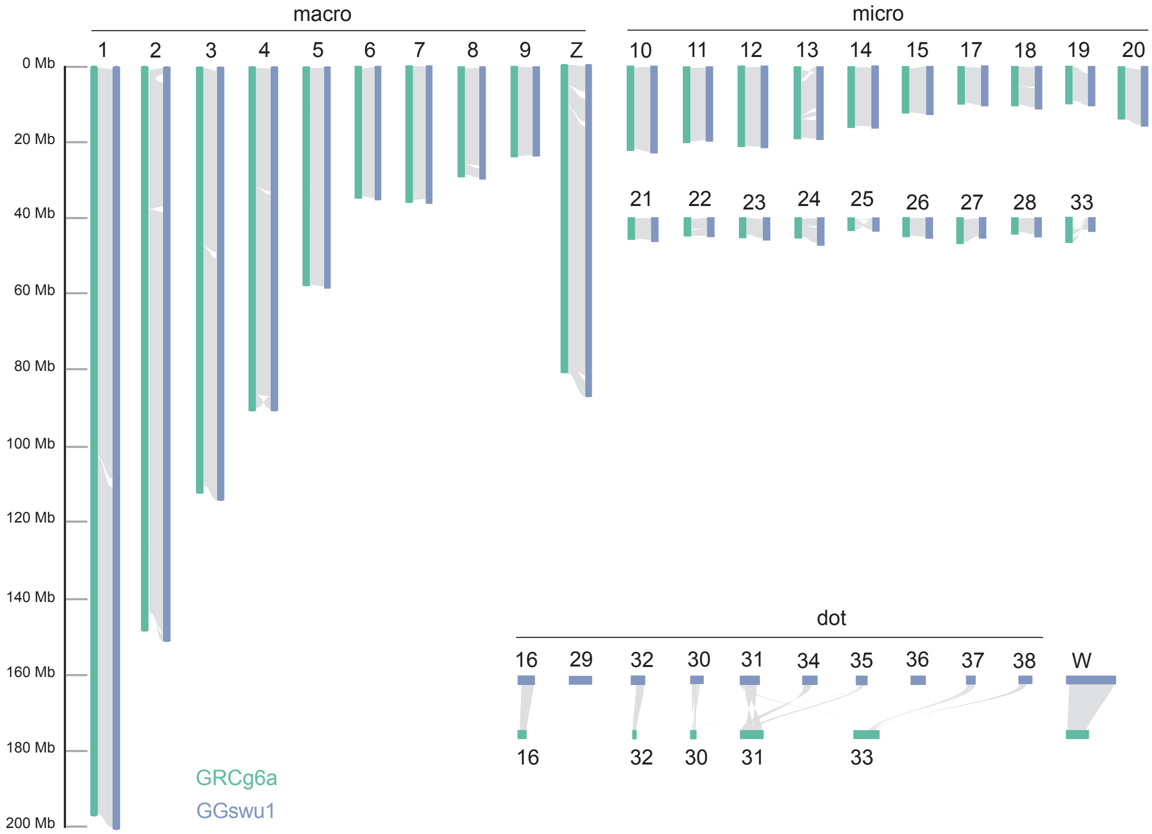


**Fig. S3. Chromosomal synteny between GRCg6a and GGsuw1.** Synteny blocks were inferred through merging orthologous pairs using MCscan. Dot chromosome synteny is shown with a different scale. No homologous genes of GGswu1 chr29 and chr36 were found in GRCg6a. GGswu1 chr31, chr34 and chr35 were found homologous with GRCg6a, while chr37 and chr38 were homologous with GRCg6a chr33.

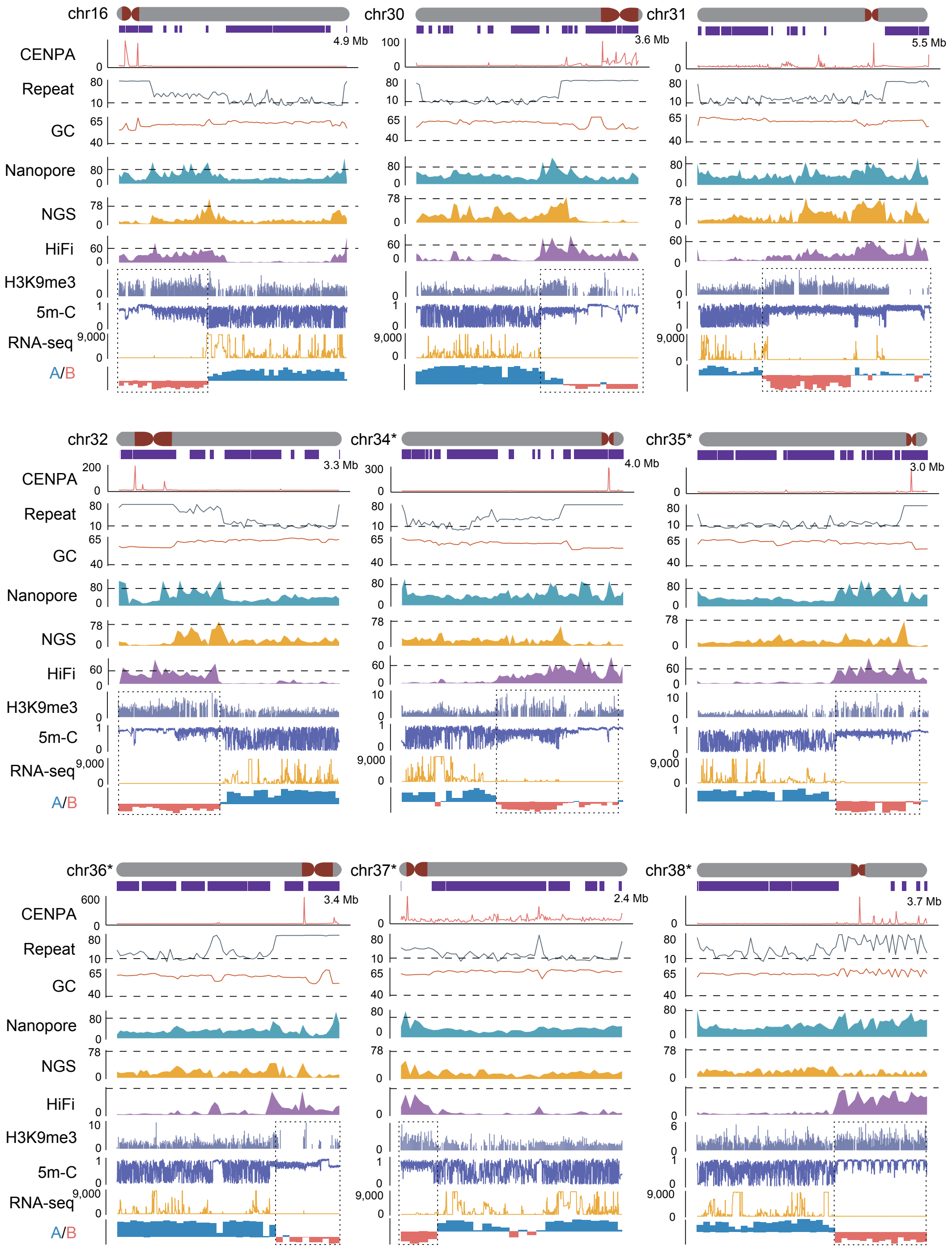


**Fig. S4. The sequences and epigenetic landscape of dot chromosomes**. The similar figure for chr29 is shown in the main figure 1. Full descriptions for the panels are also available in the main figure legend. New chromosome models were indicated by an asterisk after the chromosome names. The dashed rectangles highlighted the centromeric and pericentromeric heterochromatin.

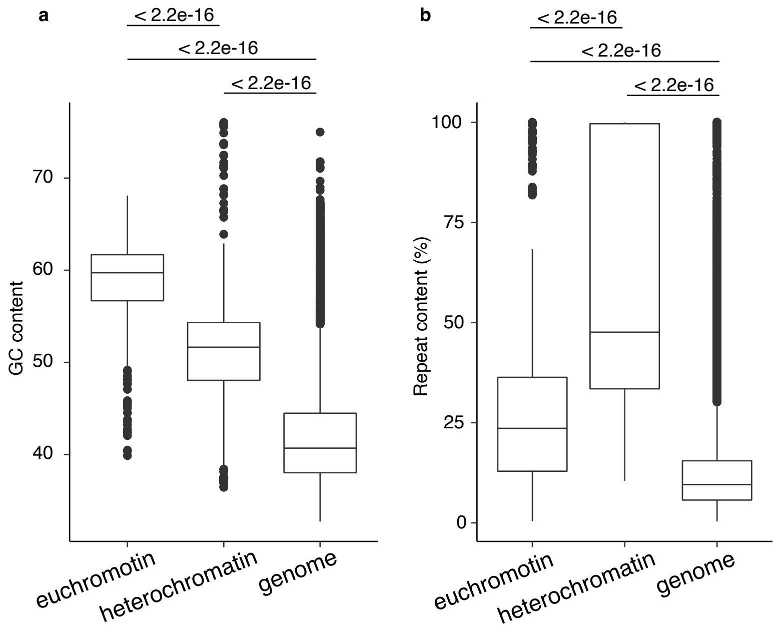


**Fig. S5. GC content and repeat content of the dot chromosomes**. Both euchromatin and heterochromatin of dot-chromosomes have significantly higher GC content and repeat content than the genomic average. The euchromatin has much higher GC content than heterochromatin, but with lower repeat content. The numbers above the boxplots indicate the p-values of Wilcoxon tests (two-tailed). The boxplots show the first quartile, median, third quartile values, and outliers.

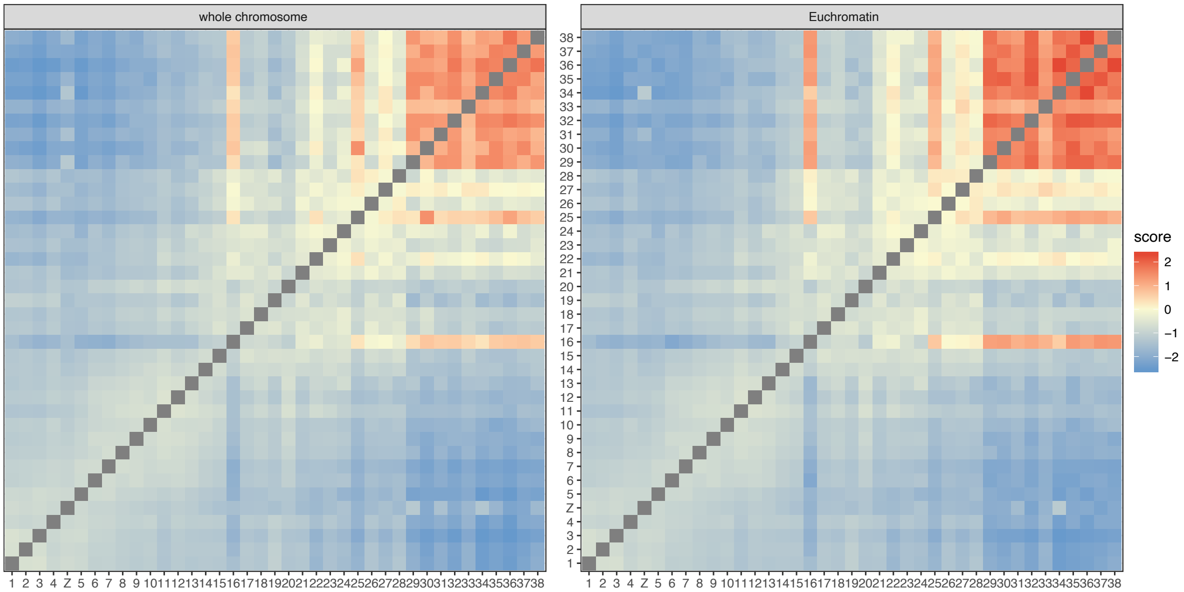


**Fig. S6. Inter-chromosomal interactions are mainly driven by euchromatin**. The intensity of interactions was measure at the chromosome level (Liu et al. 2021). Dot-chromosomes show most intensive inter-chromosomal interactions with themselves. In the right panel, heterochromatin was masked, therefore only the interactions between euchromatin were measured which showed increased intensity for dot-chromosomes.

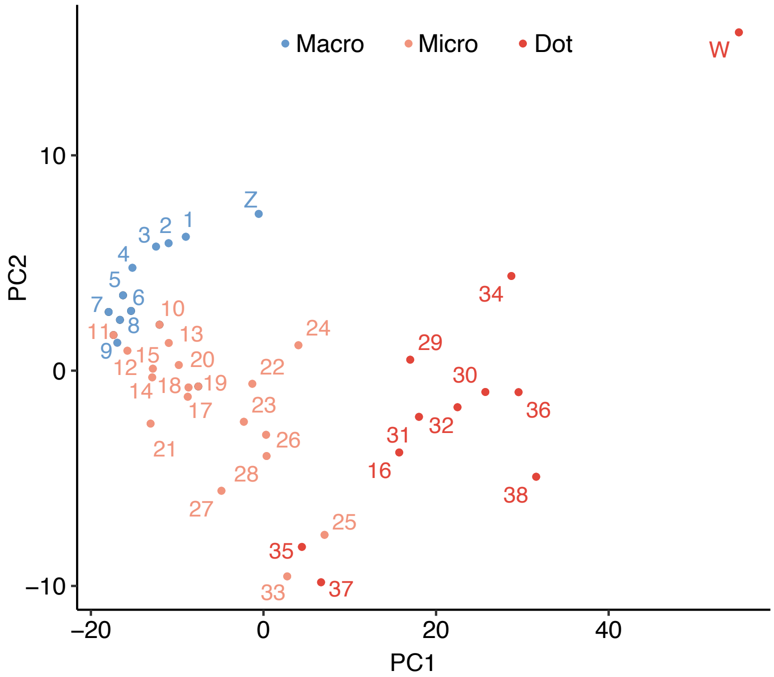


**Fig. S7. PCA analyses for sequence and epigenetic features**. Seven genomic features (shown in Fig. 1e) were used for PCA analyses. Chromosomes tend to be clustered by chromosome types. Dot-chromosomes are largely separated from both microchromosomes and macrochromosomes by PC1. chr25 and chr33 share some features with dot-chromosomes. chr11 is close to macrochromosomes and share a similar centromeric feature (Fig. S12). The W chromosome is isolated from other chromosomes.

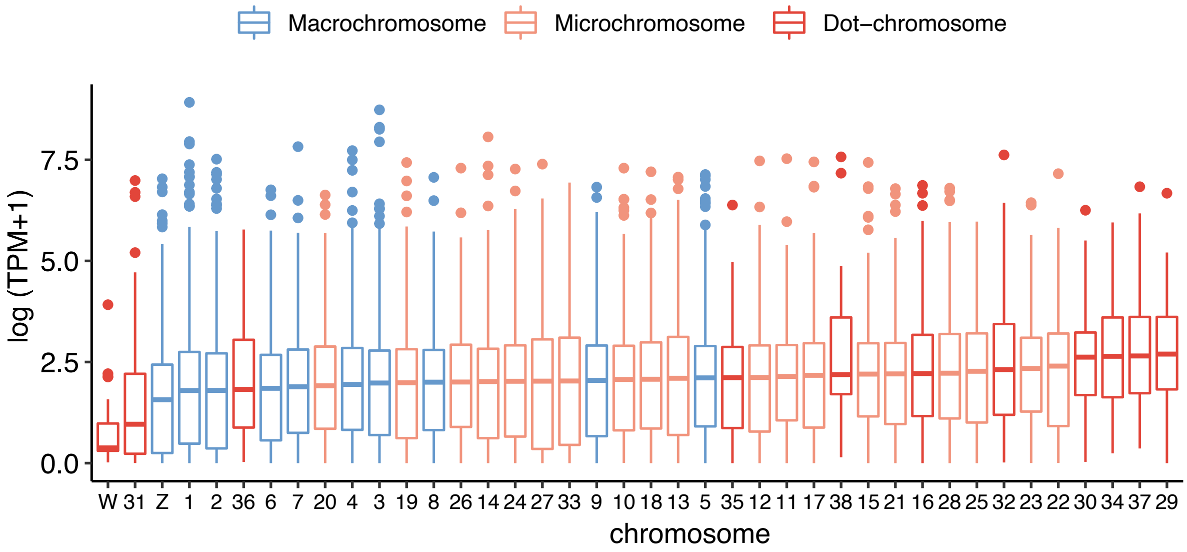


**Fig. S8. Average expression level for each chromosome**. RNA-seq data from spleens, muscles, livers, brains, hearts, lungs, kidneys, testis and ovaries were combined for account reads that were aligned to each gene model. The boxplots show the first quartile, median and third quartile values of gene expression levels for all genes in a chromosome. The W chromosome shows a low expression level partially because some samples were from males. The Z chromosome shows relative low expression in part due to a lack of complete dosage compensation in females. The dot-chromosomes tend to have a higher expression level.

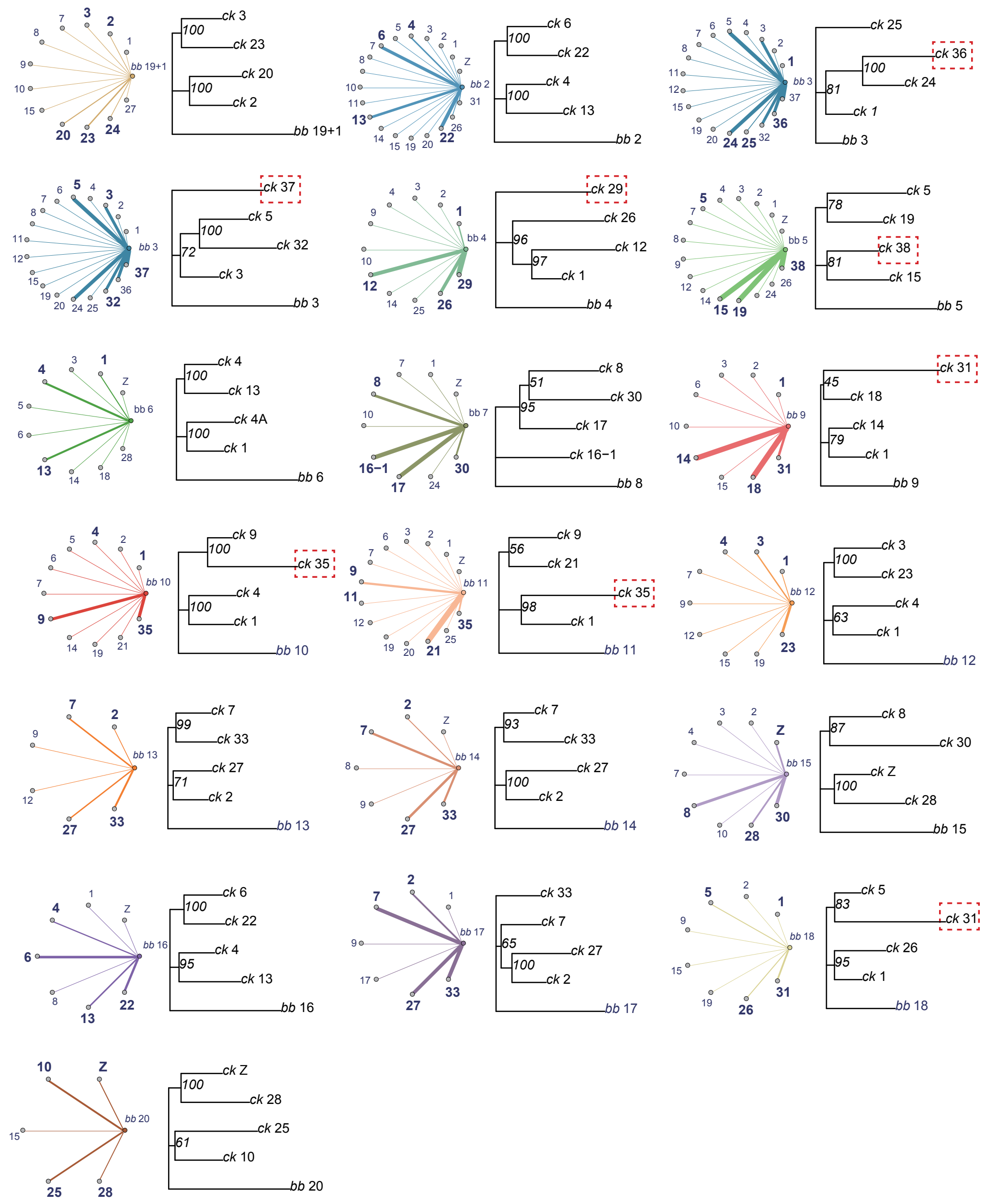

**Fig. S9. Homologous chromosome between chicken and amphioxus**. The network figures show relative proportions of orthologous genes between amphioxus (bb) and chicken (ck) chromosomes. The relative proportion (indicated by the thickness of connecting lines) for each chicken chromosome was defined as the number of orthologous genes divided by the total gene number of that chromosome. Each amphioxus chromosome shows homology with four major chicken chromosomes; in case of chromosome fusions in amphioxus (e.g. bb 3), each chromosome has eight homologous chicken chromosomes. The figure for bb 8 was shown in the main figure 2. Gene sequences from the same chromosomes were concatenated to reconstruct maximum-likelihood phylogeny for homologous chromosomes. The red dashed rectangles highlighted dot-chromosomes.

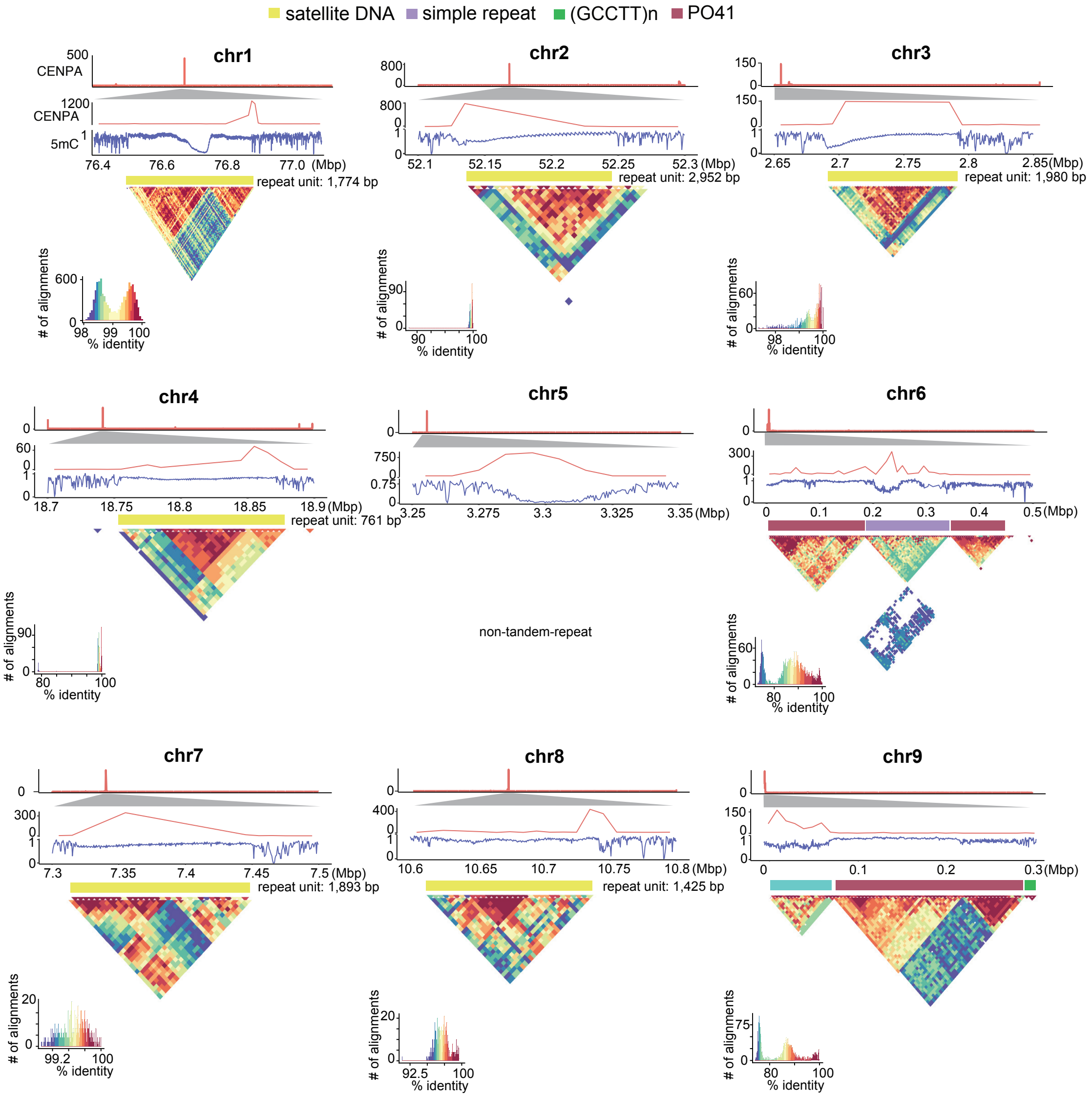


**Fig. S10. CENP-A binding, methylation and repeats of macrochromosome centromeres**. The CENP-A panel shows the ChIP/input ratio, and the 5mC panel shows 5mC levels (0 - 1) estimated with Nanopore reads. The heatmaps show the pair-wise sequence identity (%) between 4-kb sequences. Repeat units of centromeres differ by sequence and size among chromosomes. chr6 and chr9 are acrocentric whose centromeres are associated with PO41 repeats and (CCCTAA)n.

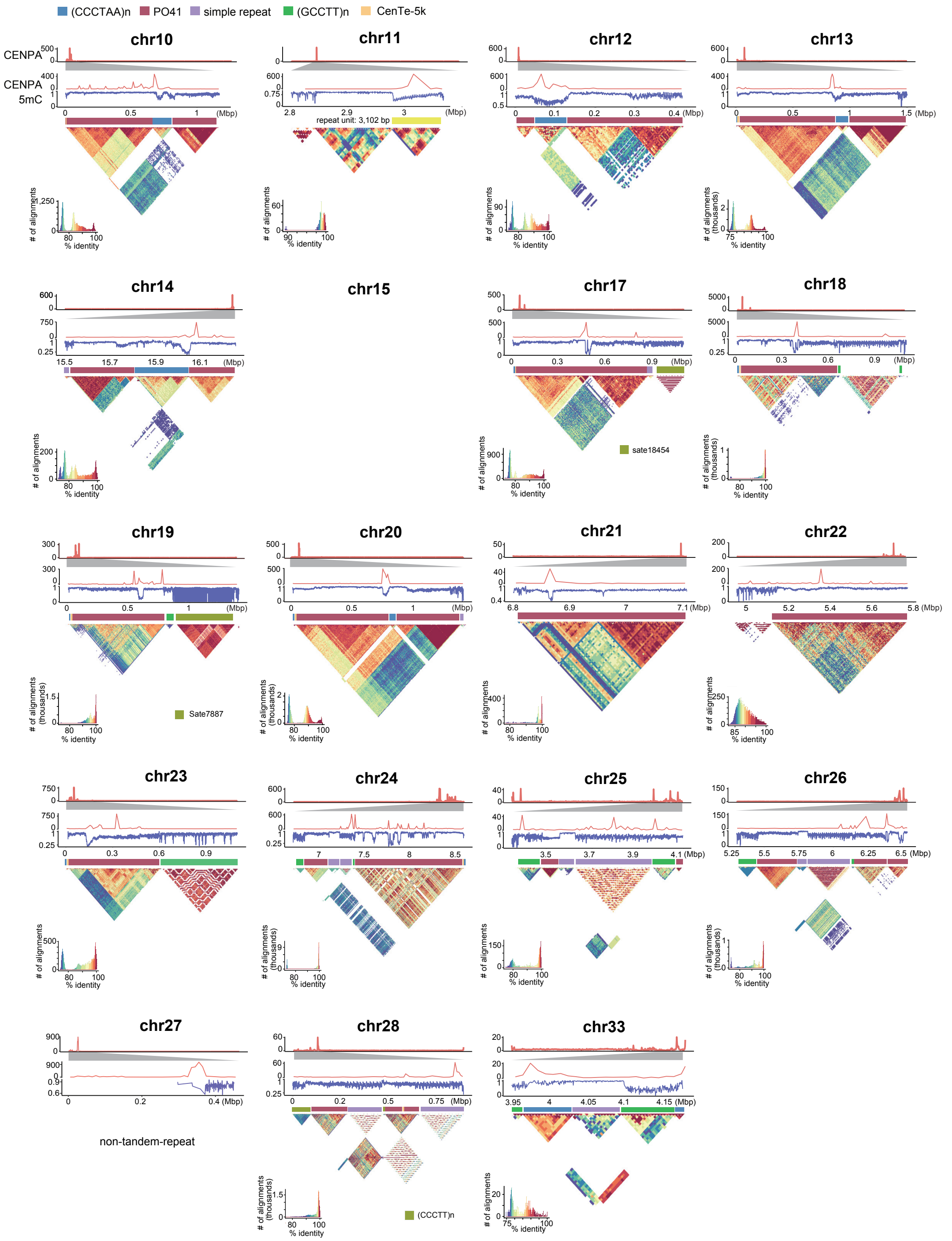

**Fig. S11. CENP-A binding, methylation and repeats of microchromosome centromeres.** The CENP-A panel shows the ChIP/input ratio, and the 5mC panel shows 5mC levels (0 - 1) estimated with Nanopore reads. The heatmaps show the pair-wise sequence identity (%) between 4-kb sequences. The repeat of chr11 centromere is unique, a characteristic typical for macrochromosomes. We failed to identify the centromeric sequence for chr15.

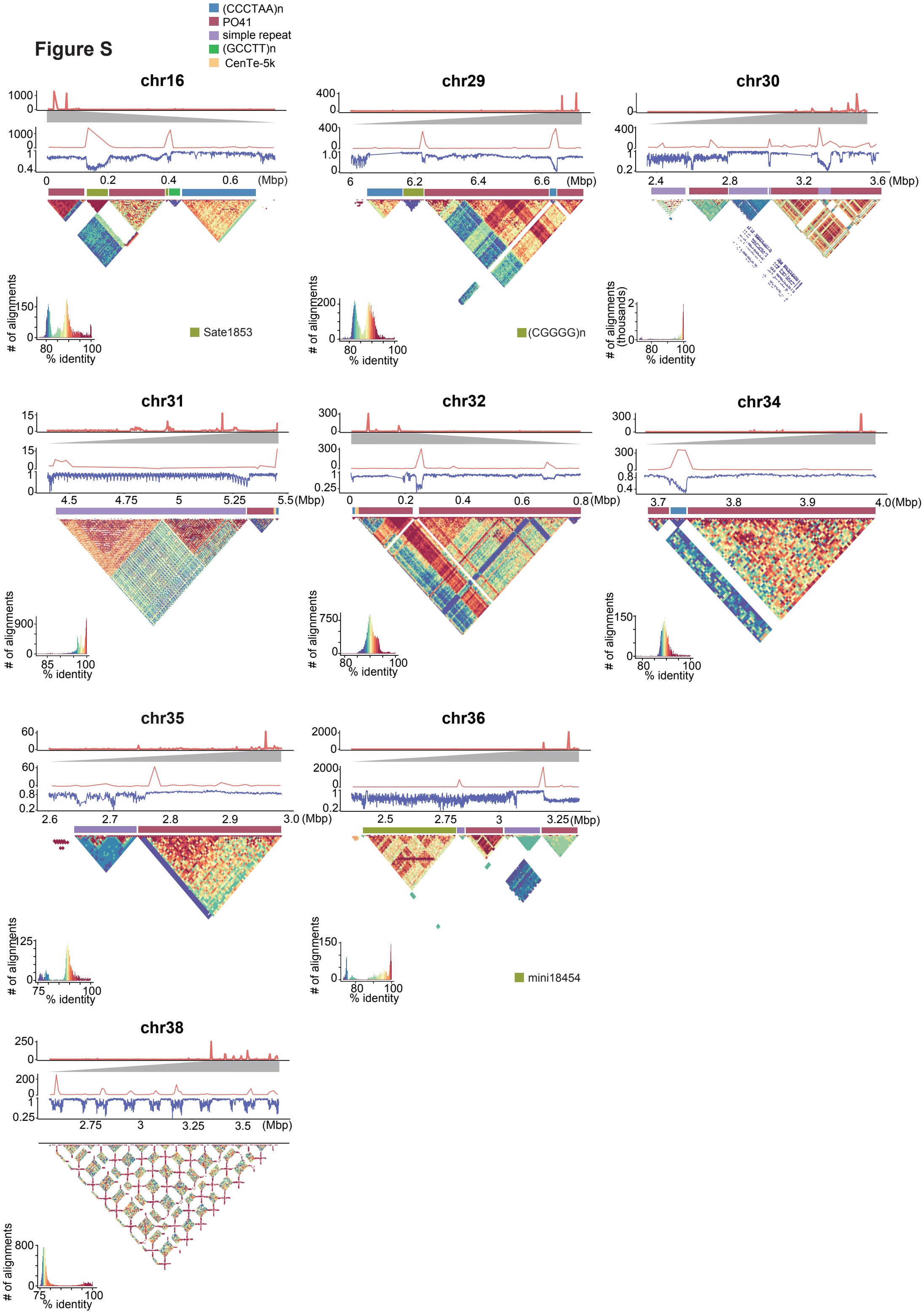
Figure S
(CCCTAA)n
PO41
simple repeat
(GCCTT)n
CenTe-5k
chr16
chr29
chr30
chr31
chr32
chr34
chr35
chr36
chr38
Sate1853
(CGGGG)n
mini18454
# of alignments
# of alignments (thousands)
% identity
(Mbp)

**Fig. S12 CENP-A binding, methylation and repeats of dot-chromosome centromeres**. The CENP-A panel shows the ChIP/input ratio, and the 5mC panel shows 5mC levels (0 - 1) estimated with Nanopore reads. The heatmaps show the pair-wise sequence identity (%) between 4-kb sequences.

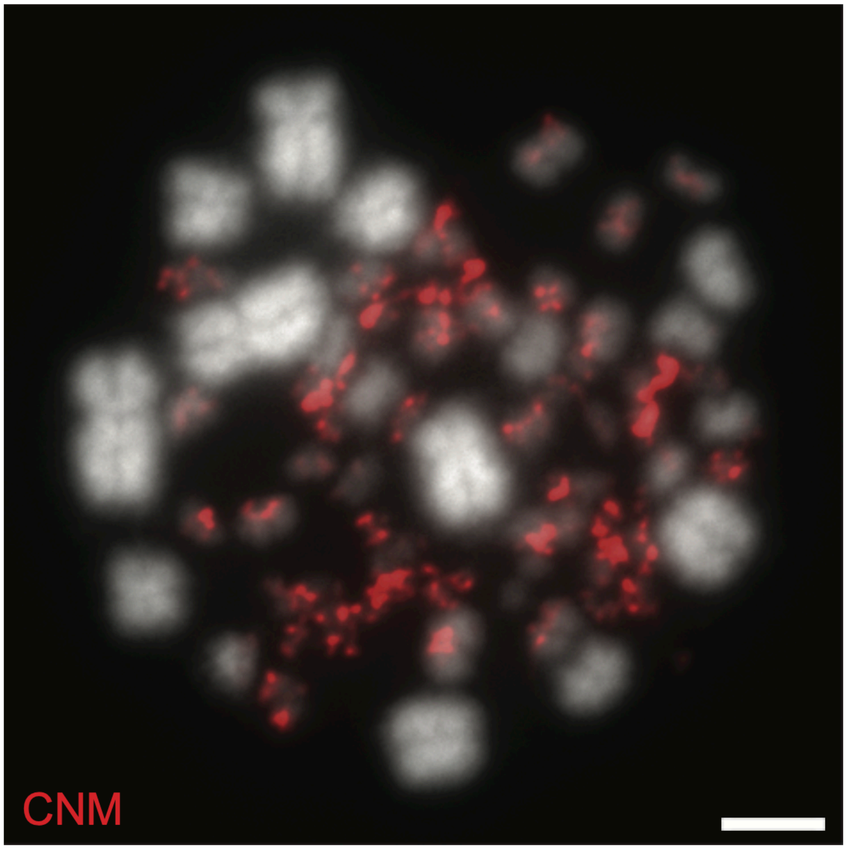


**Fig. S13 CNM repeats are abundant on acrocentric chromosomes**. The left panel shows a FISH image for PO41 binding in a chicken cell. PO41 signal appears at centromeres in acrocentric microchromosomes and dot-chromosomes. The scale for the white bar: 10μm.

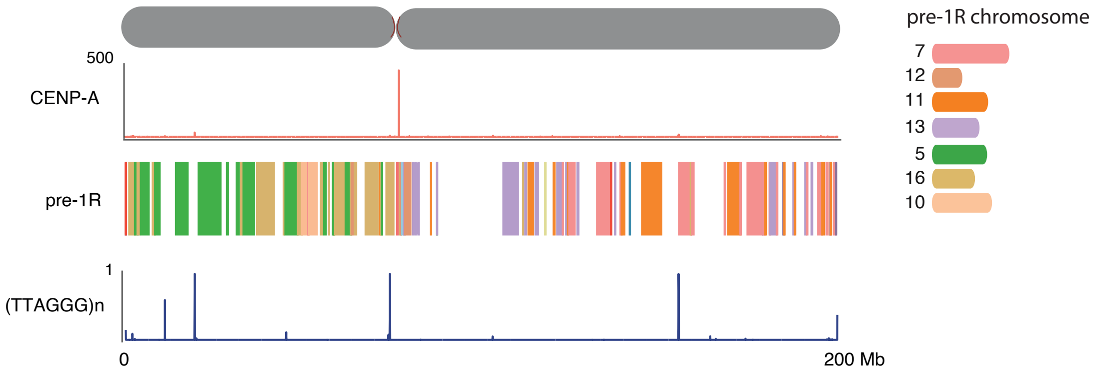


**Fig. S14 The position of CENP-A repeat on chr1 coincide with a fusion point and a large interstitial (TTAGGG)n**. The pre-1R fragments were reconstructed according their chordate origin. The density of (TTAGGG)n are calculated in 50-kb windows. The positions of interstitial (TTAGGG)s sites (ITSs) on chr1 are consistent with the FISH signal on a lampbrush chromosome from a study by Krasikova et al. (2006) (1).

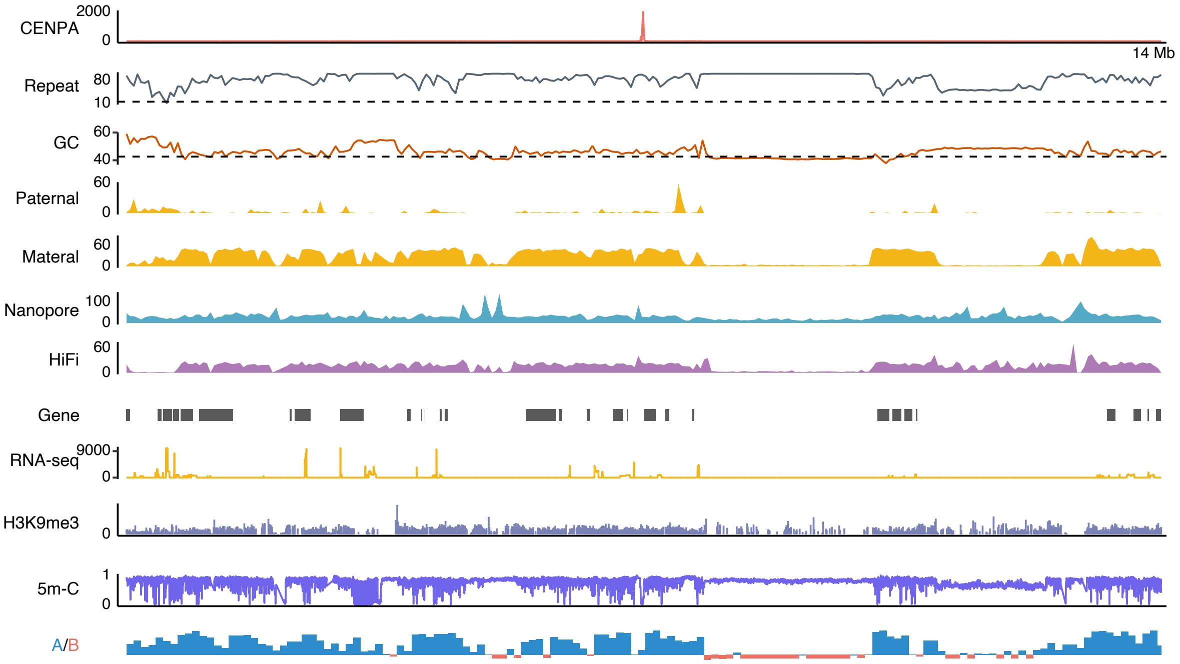


**Fig. S15 The sequence and epigenetic landscape of the W chromosome**. The description for each panel is similar to main figure 1d. Repeat content larger than 80% are shown as 80%. The NGS sequencing depth for paternal data is almost zero throughout most length of the W chromosome. The 5m-C level is extremely high except for the gene regions.

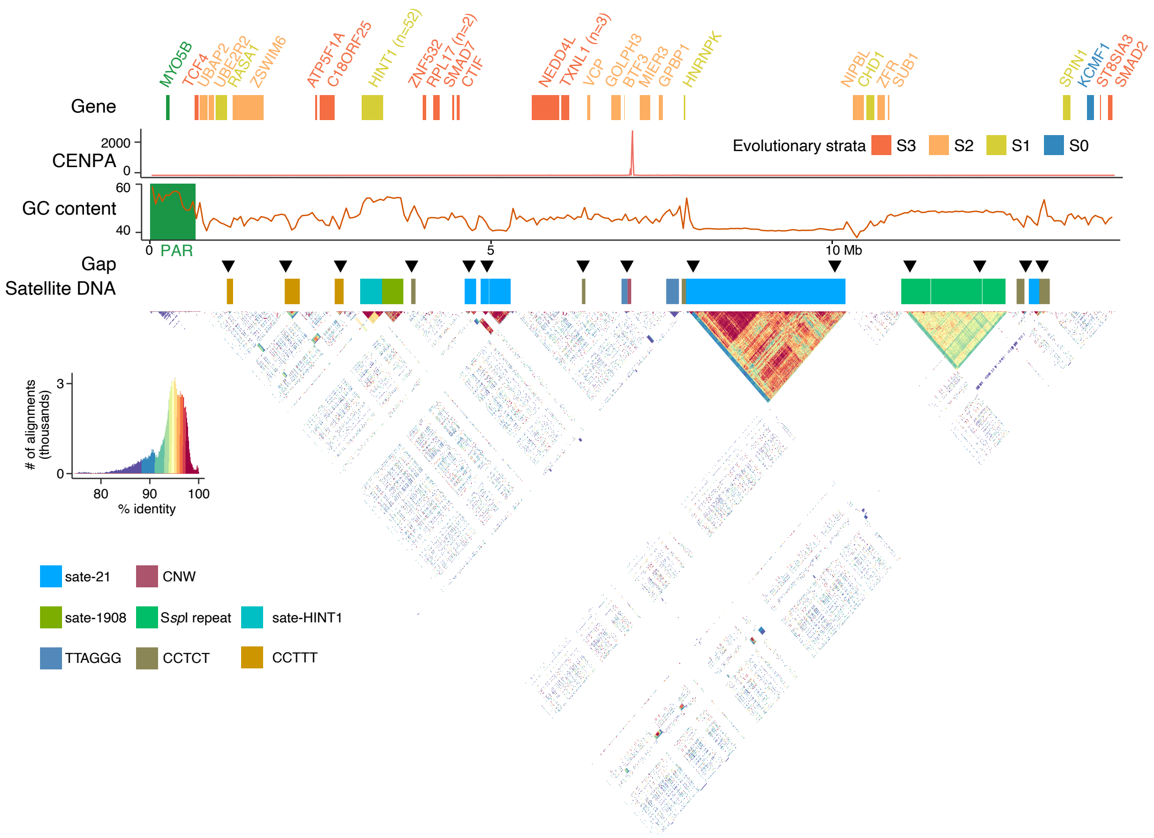


**Figure S16. The sequence and structure of chicken W chromosome.** The W chromosome contains 29 genes that are colored based on the evolutionary strata they belong to. The centromere is located in the middle with a strong CENP-A signal. The GC content is elevated on the PAR and the HINT1W amplicons. The black triangles indicate the locations of gaps that are all within satellite DNA sequences. The heatmaps show the sequence identity (%) between 4-kb sequences

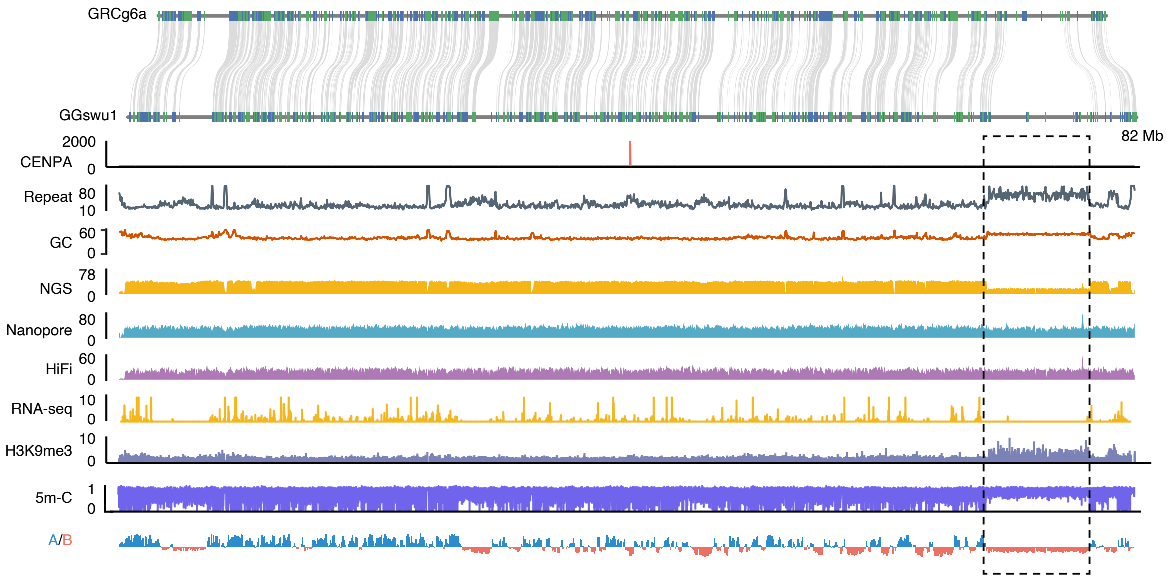


**Fig. S17 The sequence and epigenetic landscape of the Z chromosome**. The up penal show and gene synteny of the Z chromosome between GRCg6a and GGswu1. The description for each panel in the lower panel is similar to main figure 1d. A ~9 Mb hypermethylated region at the 3' end of the Z is highlighted by a black dashed rectangle.

**Table S1.** Statistics of genome assembly

| Assembly | Type | Assembly method | Dataset | Size | #contig | N50 | N90 |
|---|---|---|---|---|---|---|---|
| ont_pri | Primary | NextDenovo | ~80X Nanopore | 1,080,643,069 | 159 | 35,424,271 | 5,817,947 |
| ont_pat | Haploid 1 | NextDenovo | ~80X Nanopore | 1,049,080,350 | 178 | 17,765,433 | 2,726,683 |
| ont_mat | Haploid 2 | | | 950,862,726 | 258 | 17,952,754 | 1,707,859 |
| ont_pat_canu | Haploid 1 | canu | ~80X Nanopore | 1,041,231,341 | 465 | 14,296,215 | 1,872,337 |
| ont_mat_canu | Haploid 2 | | | 963,642,702 | 682 | 13,212,031 | 562,225 |
| hifi_pri | Primary | hifiasm | ~52X HiFi | 1,116,124,470 | 770 | 12,679,981 | 1,268,447 |
| hifi_pat | Haploid 1 | hifiasm | ~52X HiFi | 1,075,401,365 | 760 | 7,376,595 | 1,014,045 |
| hifi_mat | Haploid 2 | | | 1,007,281,665 | 806 | 7,375,302 | 997,114 |
| pri.v1 | Primary | Merging ont_pri and hifi_pri | Nanopore + HiFi | 1,078,940,082 | 66 | 87,735,852 | 11,295,715 |
| pat.final | Haploid 1 | Merging ont_pri and hifi_pat | Nanopore + HiFi | 1,058,483,130 | 68 | 87,735,852 | 10,063,281 |
| mat.final | Haploid 2 | Merging ont_pri and hifi_mat | Nanopore + HiFi | 982,619,910 | 84 | 58,898,783 | 6,729,700 |
| pri.v2 (GGswu1) | Primary | Merging pri.v1, pat.final and mat.final | Nanopore + HiFi | 1,100,555,631 | 54 | 87,735,852 | 11,295,715 |
| GRCg6a | Primary | FALCON | 82X Pacbio CLR | 1,065,348,650 | 1,402 | 17,655,422 | 2,759,626 |

**Table S2.** BUSCO completeness for chicken genome assemblies

| | Complete | | Fragmented | | Missing | |
|---|---|---|---|---|---|---|
| | **Percentage** | **Number** | **Percentage** | **Number** | **Percentage** | **Number** |
| GRCg6a | 96.9 | 8080 | 0.8 | 65 | 2.3 | 193 |
| pat.final | 96.6 | 8058 | 0.8 | 69 | 2.6 | 211 |
| mat.final | 96.7 | 8063 | 0.8 | 65 | 2.5 | 210 |
| GGswu1 | 97 | 8087 | 0.7 | 58 | 2.3 | 193 |

**Table S3.** Comparisons between GGswu1 and GRCg6a

| Chromosome | Total length | | | | GC plateaus | | | | Satellite DNA | | | |
|---|---|---|---|---|---|---|---|---|---|---|---|---|
| | GGswu1 | GRCg6a | Change (bp) | Change (%) | GGswu1 | GRCg6a | Change (bp) | Change (%) | GGswu1 | GRCg6a | Change (bp) | Change (%) |
| **chr1** | 200,044,509 | 197,608,386 | 2,436,123 | 1.23 | 3,999,920 | 1,849,963 | 2,149,957 | 116.22 | 6196335 | 4837303 | 1,359,032 | 28.09 |
| **chr2** | 152,127,819 | 149,682,049 | 2,445,770 | 1.63 | 3,599,928 | 1,349,973 | 2,249,955 | 166.67 | 3960411 | 2823201 | 1,137,210 | 40.28 |
| **chr3** | 112,377,238 | 110,838,418 | 1,538,820 | 1.39 | 499,990 | 449,991 | 49,999 | 11.11 | 3769587 | 1765491 | 2,004,096 | 113.51 |
| **chr4** | 91,364,047 | 91,315,245 | 48,802 | 0.05 | 1,599,968 | 1,399,972 | 199,996 | 14.29 | 1326270 | 1184285 | 141,985 | 11.99 |
| **chr5** | 59,473,047 | 59,809,098 | -336,051 | -0.56 | 949,981 | 699,986 | 249,995 | 35.71 | 1079338 | 874361 | 204,977 | 23.44 |
| **chr6** | 36,158,469 | 36,374,701 | -216,232 | -0.59 | 549,989 | 499,990 | 49,999 | 10.00 | 1430776 | 933251 | 497,525 | 53.31 |
| **chr7** | 36,439,816 | 36,742,308 | -302,492 | -0.82 | 599,988 | 649,987 | -49,999 | -7.69 | 509116 | 365132 | 143,984 | 39.43 |
| **chr8** | 29,594,148 | 30,219,446 | -625,298 | -2.07 | 499,990 | 499,990 | 0 | 0.00 | 574690 | 712021 | -137,331 | -19.29 |
| **chr9** | 23,966,833 | 24,153,086 | -186,253 | -0.77 | 599,988 | 749,985 | -149,997 | -20.00 | 569533 | 396426 | 173,107 | 43.67 |
| **chr10** | 21,606,253 | 21,119,840 | 486,413 | 2.30 | 849,983 | 949,981 | -99,998 | -10.53 | 1486107 | 673234 | 812,873 | 120.74 |
| **chr11** | 19,822,308 | 20,200,042 | -377,734 | -1.87 | 1,099,978 | 1,049,979 | 49,999 | 4.76 | 499015 | 229748 | 269,267 | 117.20 |
| **chr12** | 20,591,337 | 20,387,278 | 204,059 | 1.00 | 949,981 | 799,984 | 149,997 | 18.75 | 774071 | 707680 | 66,391 | 9.38 |
| **chr13** | 19,253,982 | 19,166,714 | 87,268 | 0.46 | 849,983 | 1,149,977 | -299,994 | -26.09 | 1445341 | 395450 | 1,049,891 | 265.49 |
| **chr14** | 16,259,740 | 16,219,308 | 40,432 | 0.25 | 1,499,970 | 1,449,971 | 49,999 | 3.45 | 712821 | 560867 | 151,954 | 27.09 |
| **chr15** | 13,529,386 | 13,062,184 | 467,202 | 3.58 | 499,990 | 499,990 | 0 | 0.00 | 933070 | 294326 | 638,744 | 217.02 |
| **chr16** | 4,857,080 | 2,844,601 | 2,012,479 | 70.75 | 2,649,947 | 699,986 | 1,949,961 | 278.57 | 930023 | 447012 | 483,011 | 108.05 |
| **chr17** | 11,322,113 | 10,762,512 | 559,601 | 5.20 | 899,982 | 799,984 | 99,998 | 12.50 | 1103587 | 102700 | 1,000,887 | 974.57 |
| **chr18** | 12,199,395 | 11,373,140 | 826,255 | 7.26 | 1,249,975 | 1,349,973 | -99,998 | -7.41 | 1252541 | 313553 | 938,988 | 299.47 |
| **chr19** | 11,295,715 | 10,323,212 | 972,503 | 9.42 | 1,299,974 | 999,980 | 299,994 | 30.00 | 1276920 | 296223 | 980,697 | 331.07 |
| **chr20** | 15,361,164 | 13,897,287 | 1,463,877 | 10.53 | 999,980 | 899,982 | 99,998 | 11.11 | 1579451 | 263679 | 1,315,772 | 499.01 |
| **chr21** | 7,106,554 | 6,844,979 | 261,575 | 3.82 | 499,990 | 649,987 | -149,997 | -23.08 | 399064 | 138241 | 260,823 | 188.67 |
| **chr22** | 5,772,503 | 5,459,462 | 313,041 | 5.73 | 1,399,972 | 1,199,976 | 199,996 | 16.67 | 898823 | 221965 | 676,858 | 304.94 |
| **chr23** | 6,970,121 | 6,149,580 | 820,541 | 13.34 | 1,399,972 | 1,449,971 | -49,999 | -3.45 | 1075216 | 259785 | 815,431 | 313.89 |
| **chr24** | 8,591,846 | 6,491,222 | 2,100,624 | 32.36 | 849,983 | 549,989 | 299,994 | 54.55 | 1548552 | 134814 | 1,413,738 | 1048.66 |
| **chr25** | 4,095,597 | 3,980,610 | 114,987 | 2.89 | 2,699,946 | 1,949,961 | 749,985 | 38.46 | 652240 | 378439 | 273,801 | 72.35 |
| **chr26** | 6,534,586 | 6,055,710 | 478,876 | 7.91 | 1,499,970 | 1,549,969 | -49,999 | -3.23 | 1026360 | 110265 | 916,095 | 830.81 |
| **chr27** | 6,322,964 | 8,080,432 | -1,757,468 | -21.75 | 2,149,957 | 2,049,959 | 99,998 | 4.88 | 337591 | 1987279 | -1,649,688 | -83.01 |
| **chr28** | 5,942,585 | 5,116,882 | 825,703 | 16.14 | 2,199,956 | 1,949,961 | 249,995 | 12.82 | 859289 | 239750 | 619,539 | 258.41 |
| **chr29*** | 6,729,861 | | 6,729,861 | | 2,149,957 | | 2,149,957 | | 1199692 | | 1,199,692 | |
| **chr30** | 3,608,281 | 1,818,525 | 1,789,756 | 98.42 | 2,299,954 | 1,499,970 | 799,984 | 53.33 | 1154260 | 247400 | 906,860 | 366.56 |
| **chr31** | 5,465,193 | 6,153,034 | -687,841 | -11.18 | 2,549,949 | 999,980 | 1,549,969 | 155.00 | 1285156 | 999976 | 285,180 | 28.52 |
| **chr32** | 3,259,096 | 725,831 | 2,533,265 | 349.02 | 2,199,956 | 649,987 | 1,549,969 | 238.46 | 1333252 | 34771 | 1,298,481 | 3734.38 |
| **chr33** | 4,180,360 | 7,821,666 | -3,641,306 | -46.55 | 2,949,941 | 3,299,934 | -349,993 | -10.61 | 220588 | 1017999 | -797,411 | -78.33 |
| **chr34*** | 3,990,469 | | 3,990,469 | | 1,049,979 | | 1,049,979 | | 1467366 | | 1,467,366 | |
| **chr35*** | 2,985,734 | | 2,985,734 | | 2,199,956 | | 2,199,956 | | 530569 | | 530,569 | |
| **chr36*** | 3,355,600 | | 3,355,600 | | 2,449,951 | | 2,449,951 | | 1155590 | | 1,155,590 | |
| **chr37*** | 2,448,051 | | 2,448,051 | | 1,999,960 | | 1,999,960 | | 314584 | | 314,584 | |
| **chr38*** | 3,669,724 | | 3,669,724 | | 3,249,935 | | 3,249,935 | | 508610 | | 508,610 | |
| **chrW** | 14,151,827 | 6,813,114 | 7,338,713 | 107.71 | 349,993 | 0 | 349,993 | | 5136630 | 449136 | 4,687,494 | |
| **chrZ** | 87,735,852 | 82,529,921 | 5,205,931 | 6.31 | 1,699,966 | 799,984 | 899,982 | 112.50 | 8231749 | 5248964 | 2,982,785 | 56.83 |
| **Total** | 1,100,561,203 | 1,050,139,823 | 50,421,380 | 4.80 | 63,598,728 | 37,399,252 | 26,199,476 | 70.05 | 60,744,184 | 29,644,727 | 31,099,457 | 104.91 |

**Table S4.** Chromosome model and size comparison

| GGswu1 | | GRCg7b | | Change | Change (%) |
|---|---|---|---|---|---|
| **Chr.** | **Size** | **Chr.** | **Size** | | |
| chr16 | 4,857,080 | chr16 | 2,706,039 | 2,151,041 | 79.5 |
| chr29 | 6,729,861 | chr33 | 3,839,931 | 2,889,930 | 75.3 |
| chr30 | 3,608,281 | chr30 | 755,666 | 2,852,615 | 377.5 |
| chr31 | 5,465,193 | chr31 | 2,457,334 | 3,007,859 | 122.4 |
| chr32 | 3,259,096 | chr32 | 125,424 | 2,466,360 | 311.1 |
| | | chr38 | 667,312 | | |
| chr34 | 3,990,469 | chr29 | 726,478 | 2,709,865 | 211.6 |
| | | chr35 | 554,126 | | |
| chr35 | 2,985,734 | chr37 | 786,586 | 2,199,148 | 279.6 |
| chr36 | 3,355,600 | NA | NA | NA | NA |
| chr37 | 2,448,051 | chr39 | 157,853 | 2,290,198 | 1450.8 |
| chr38 | 3,669,724 | chr38 | 667,312 | 3,002,412 | 449.9 |
| Total | 40,369,089 | Total | 13,444,061 | 26,925,028 | 200.3 |

**Table S5.** Mapping BAC clones to two chicken assemblies

| | GGswu1 | GRCg7b |
|---|---|---|
| Aligned Bases | 199,478,444 | 199,162,469 |
| Aligned rate | 99.85% | 99.69% |
| 1-to-1 alignment average identity | 99.22 | 99.21 |

**Table S6.** Summary of missing sequences of dot chromosomes in GRCg6a

| | Heterochromatin | | | Euchromatin | | |
|---|---|---|---|---|---|---|
| | **Size** | **Missing in GRCg6a** | **Missing %** | **Size** | **Missing in GRCg6a** | **Missing %** |
| chr16 | 1,950,000 | 909,259 | 46.63 | 2,866,001 | 2,184,211 | 76.21 |
| chr29 | 4,450,000 | 1,088,544 | 24.46 | 2,248,000 | 1,941,571 | 86.37 |
| chr30 | 1,150,000 | 931,992 | 81.04 | 2,413,000 | 1,046,855 | 43.38 |
| chr31 | 1,900,000 | 354,553 | 18.66 | 3,505,001 | 2,498,946 | 71.30 |
| chr32 | 1,500,000 | 1,083,577 | 72.24 | 1,731,001 | 1,092,504 | 63.11 |
| chr34 | 2,200,000 | 1,192,767 | 54.22 | 1,728,000 | 1,521,174 | 88.03 |
| chr35 | 1,150,000 | 881,288 | 76.63 | 1,788,000 | 1,630,833 | 91.21 |
| chr36 | 950,000 | 738,168 | 77.70 | 2,395,000 | 2,133,489 | 89.08 |
| chr37 | 650,000 | 305,946 | 47.07 | 1,667,002 | 1,287,535 | 77.24 |
| chr38 | 1,400,000 | 231,703 | 16.55 | 2,222,000 | 2,177,515 | 98.00 |
| Total | 17,300,000 | 7,717,797 | 44.61 | 22,563,005 | 17,514,633 | 77.63 |

**Table S7.** Genomic property for each chicken chromosome

| chromosome | GC content | Size | H3K27me3 | H3K9me3 | H3K36me3 | 5-mC | repeat content |
|---|---|---|---|---|---|---|---|
| Z | 41.59 | 87,735,852 | 2.91 | 1.05 | 0.266 | 0.669 | 24.9 |
| 1 | 40.65 | 200,044,509 | 1.45 | 1.04 | 0.248 | 0.600 | 16.4 |
| 2 | 40.4 | 152,127,819 | 1.58 | 1.02 | 0.341 | 0.596 | 14.4 |
| 3 | 40.14 | 112,377,238 | 1.52 | 1.02 | 0.267 | 0.564 | 13.0 |
| 4 | 40.39 | 91,364,047 | 1.66 | 1.03 | 0.276 | 0.555 | 10.1 |
| 5 | 41.3 | 59,473,047 | 1.55 | 1.06 | 0.333 | 0.542 | 8.7 |
| 6 | 42.16 | 36,158,469 | 1.70 | 1.16 | 0.323 | 0.568 | 9.4 |
| 7 | 41.56 | 36,439,816 | 1.71 | 1.15 | 0.324 | 0.567 | 6.9 |
| 8 | 42.21 | 29,594,148 | 1.56 | 1.14 | 0.405 | 0.565 | 8.1 |
| 9 | 43.14 | 23,966,833 | 1.75 | 1.23 | 0.597 | 0.547 | 7.5 |
| 10 | 43.52 | 21,606,253 | 1.63 | 1.23 | 0.421 | 0.569 | 12.5 |
| 11 | 42.65 | 19,822,308 | 1.70 | 1.16 | 0.415 | 0.576 | 7.2 |
| 12 | 43.77 | 20,591,337 | 1.63 | 1.27 | 0.399 | 0.584 | 8.6 |
| 13 | 44.6 | 19,253,982 | 2.01 | 1.40 | 0.418 | 0.532 | 13.3 |
| 14 | 45.66 | 16,259,740 | 1.54 | 1.34 | 0.607 | 0.654 | 11.0 |
| 15 | 45.25 | 13,529,386 | 1.66 | 1.34 | 0.683 | 0.520 | 11.2 |
| 20 | 45.86 | 15,361,164 | 2.04 | 1.44 | 0.476 | 0.614 | 14.2 |
| 17 | 47.51 | 11,322,113 | 1.88 | 1.54 | 0.577 | 0.588 | 14.8 |
| 18 | 47.12 | 12,199,395 | 1.85 | 1.46 | 0.530 | 0.554 | 15.0 |
| 19 | 47.34 | 11,295,715 | 1.59 | 1.46 | 0.766 | 0.507 | 16.1 |
| 21 | 47.58 | 7,106,554 | 1.62 | 1.47 | 0.714 | 0.534 | 10.3 |
| 22 | 48.67 | 5,772,503 | 1.90 | 1.56 | 0.551 | 0.621 | 22.2 |
| 23 | 50.19 | 6,970,121 | 2.13 | 1.65 | 0.729 | 0.536 | 20.9 |
| 24 | 48.3 | 8,591,846 | 2.19 | 1.78 | 0.934 | 0.622 | 27.9 |
| 26 | 51.42 | 6,534,586 | 2.36 | 1.71 | 0.598 | 0.548 | 23.2 |
| 27 | 52.68 | 6,322,964 | 2.59 | 1.76 | 0.671 | 0.415 | 17.6 |
| 28 | 52.38 | 5,942,585 | 1.60 | 1.60 | 0.851 | 0.486 | 23.0 |
| 25 | 57.59 | 4,096,356 | 1.61 | 1.82 | 0.733 | 0.540 | 28.6 |
| 33 | 58.42 | 4,181,167 | 2.36 | 1.87 | 0.607 | 0.589 | 24.0 |
| 16 | 56 | 4,857,269 | 0.41 | 1.93 | 0.167 | 0.469 | 37.9 |
| 29 | 52.13 | 6,729,691 | 0.34 | 2.07 | 0.094 | 0.781 | 40.2 |
| 30 | 55.67 | 3,608,281 | 0.49 | 2.03 | 0.698 | 0.673 | 48.3 |
| 31 | 54.96 | 5,465,193 | 0.46 | 2.11 | 0.123 | 0.723 | 40.5 |
| 32 | 55.55 | 3,259,494 | 0.24 | 1.96 | 0.094 | 0.778 | 45.0 |
| 34 | 51.15 | 3,990,469 | 0.21 | 1.81 | 0.111 | 0.702 | 52.5 |
| 35 | 57.42 | 2,985,734 | 0.27 | 1.89 | 0.144 | 0.755 | 25.9 |
| 36 | 56.64 | 3,355,600 | 0.37 | 1.82 | 0.130 | 0.703 | 52.0 |
| 37 | 59.56 | 2,448,051 | 0.25 | 1.75 | 0.261 | 0.557 | 27.6 |
| 38 | 61.03 | 3,669,724 | 0.23 | 1.86 | 0.104 | 0.844 | 53.0 |
| W | 46.75 | 14,151,827 | 0.02 | 1.83 | 0.170 | 0.796 | 80.9 |

**Table S8.** Location of the CenTe-5k on each chromosome

| Chromosome | 5 ‘prime | | 3’ prime | |
|---|---|---|---|---|
| | Start | End | Start | End |
| chr1 | 8542 | 13721 | 200024844 | 200029779 |
| chr2 | 6751 | 11665 | 152105865 | 152111083 |
| chr3 | 12549 | 17764 | 112351934 | 112357217 |
| chr4 | | | 91338200 | 91346201 |
| chr5 | 8284 | 12538 | | |
| chr10 | 2834 | 8066 | | |
| chr11 | 11192 | 16138 | | |
| chr13 | 13506 | 18741 | | |
| chr16 | | | 4837758 | 4842722 |
| chr17 | 4858 | 10093 | 11303304 | 11305469 |
| chr18 | 9205 | 14598 | | |
| chr19 | 10219 | 14240 | 11277766 | 11280869 |
| chr20 | 13765 | 18703 | | |
| chr21 | 15494 | 21850 | | |
| chr22 | 17322 | 22245 | | |
| chr23 | 14258 | 19496 | 6946729 | 6951714 |
| chr24 | 5488 | 9600 | 8582337 | 8586322 |
| chr25 | 10639 | 15600 | | |
| chr26 | 9536 | 14861 | | |
| chr28 | | | 5925029 | 5930261 |
| chr29 | 9523 | 14479 | | |
| chr30 | 8180 | 13140 | | |
| chr31 | 4998 | 9954 | 5447380 | 5452339 |
| chr32 | 15490 | 22096 | | |
| chr33 | 19889 | 24811 | | |
| chr35 | 14316 | 19277 | | |
| chr38 | 20184 | 25392 | | |
| chrZ | | | 87724587 | 87729856 |

**Table S9.** Sequencing data from SRA or this study

| Accession | Sequencing type | Sample | Source |
|---|---|---|---|
| SRX9936539 | resequencing | Paternal blood | This study |
| SRX9936540 | resequencing | Maternal blood | This study |
| SRX11722311 | HiFi | F1 chick muscle | This study |
| SRX11722310 | HiFi | F1 chick muscle | This study |
| SRX11722312 | HiFi | F1 chick muscle | This study |
| SRX11722867 | Nanopore ultra-long | F1 chick muscle | This study |
| SRX11722868 | Nanopore ultra-long | F1 chick muscle | This study |
| SRX11722869 | Nanopore ultra-long | F1 chick muscle | This study |
| SRX11722871 | Nanopore ultra-long | F1 chick muscle | This study |
| SRX11722870 | Nanopore ultra-long | F1 chick muscle | This study |
| SRX11725011 | Hi-C | F1 chick muscle | This study |
| SRR18788786 | Nanopore RNA-seq | F1 chick brain | This study |
| SRR18788785 | Nanopore RNA-seq | F1 chick spleen | This study |
| SRR18788904 | H3K9me3 ChIP-seq | Liver | This study |
| SRR18788903 | Input ChIP-seq | Liver | This study |
| SRR18788845 | RNA-seq | F1 chick brain | This study |
| SRR18788807 | RNA-seq | F1 chick heart | This study |
| SRR18788805 | RNA-seq | F1 chick liver | This study |
| SRR18788806 | RNA-seq | F1 chick muscle | This study |
| SRR18788844 | RNA-seq | F1 chick spleen | This study |
| DRR018430 | CENP-A ChIP-seq | wild-type cells | Hori et al. 2014 |
| SRR12697592 | H3K27me3 ChIP-seq | cerebral cortex | Kern et al. 2021 |
| SRR15150478 | H3K36me3 ChIP-seq | DT40 | Tarsalainen et al. 2022 |
| PRJNA488330 | PacBio Iso-Seq | embryo | Ren et al. 2019 |
| ERR753952 | RNA-seq | embryonic kidney | Uebbing et al. 2015 |
| SRR10161493 | RNA-seq | adult ovary | Li et al. 2022 |
| SRR10161501 | RNA-seq | adult testis | Li et al. 2022 |
| SRR10161500 | RNA-seq | adult hypothalamus | Li et al. 2022 |
| SRR10161498 | RNA-seq | adult hypophysis | Li et al. 2022 |
| SRR10161495 | RNA-seq | adult spleen | Li et al. 2022 |
| SRR10161499 | RNA-seq | adult muscle | Li et al. 2022 |
| SRR10161497 | RNA-seq | adult liver | Li et al. 2022 |

**Dataset S1 (separate file).** Ohnolog group across amphioxus and vertebrate.

**Dataset S2 (separate file).** Oligonucleotides synthesized across the euchromatin of dot chromosomes.

### SI References

1. A. Krasikova, *et al.*, On the positions of centromeres in chicken lampbrush chromosomes. *Chromosome Res* **14**, 777–789 (2006).
